# Intragroup and Intergroup Pairwise Key Predistribution for Wireless Sensor Networks

**DOI:** 10.3390/s25010086

**Published:** 2024-12-26

**Authors:** Ching-Nung Yang, Ting-Song Gu, Jhou-Cian You, Chang-Ji Wang

**Affiliations:** 1 Department of Computer Science and Information Engineering, National Dong Hwa University, Hualien 974301, Taiwan; 611021230@gms.ndhu.edu.tw (T.-S.G.); 611221253@gms.ndhu.edu.tw (J.-C.Y.); 2 School of Information Science and Technology, Guangdong University of Foreign Studies, Guangzhou 510420, China; wchangji@126.com

**Keywords:** wireless sensor network, multiple-sink WSN (MWSN), key pre-distribution, hash chain node capture attack

## Abstract

The major task of a wireless sensor network (WSN) is data collection. Key predistribution (KP) is to establish pairwise keys for secure communication in a WSN, such that all collected data could be securely sent to a backend database. Most research on KP-like schemes is dedicated to enhancing resiliency against node capture attack (NA) and retaining the link connectivity in the meantime. For large-scale wireless sensor networks, a more common approach is to use a multiple-sink WSN (MWSN) to support a large number of sensor nodes. In MWSNs, there are different clusters (referred to as groups). We took the lead in studying KP in the MWSN environment. Based on the new MWSN environment, we present intragroup and intergroup KP (I^2^KP) to fulfill both requirements of security and energy efficiency when gathering data via various sink nodes in a large-scale WSN. Three types of I^2^KP with respective pros and cons are proposed. Theoretical analysis and numerical simulation demonstrate their effectiveness.

## 1. Introduction

A wireless sensor network (WSN) is a low-cost, short-distance, and easy-to-deploy wireless network architecture that is widely used in in various applications. For example, it can be applied to agricultural environments, smart metering in power transmission systems, and the Internet of Things [1,2,3,4]. The above applications basically require secure communication. Furthermore, security issues will become even more important if a WSN is deployed for certain mission-critical applications. Therefore, secure links must be established between sensor nodes to ensure data security and privacy in wireless sensor networks. In general, there are two types of key management protocols to secure communications in WSNs. One is pairwise key distribution to share a pairwise key between two sensor nodes [5,6,7,8,9,10,11,12,13,14,15], and the other is group key distribution to share a conference key for the sensor nodes in group communication [16,17,18]. As introduced in [5], key predistribution (KP) is firstly proposed for sharing a pairwise key between two neighbored nodes, where each node is pre-loaded with several keys selected from a key pool. There are two types of KP approaches: one is the random KP (RKP) [5,10,11,15] and the other is the deterministic KP (DKP) [8,9,14]. Generally, RKP uses light-weight cryptography, but DKP guarantees connectivity. Hybrid schemes [19] combined with RKP and DKP have both deterministic and probabilistic properties.

To enhance resiliency against node capture attacks (NAs), during which attackers capture and compromise nodes to collect keys for eavesdropping on communications among other uncompromised nodes, *q*-composite KP and hash chain-based KP (HKP) were accordingly proposed to resist NAs. However, these two approaches have their pros and cons. *q*-composite KP [12,13] does not need hash operation but reduces connectivity. On the other hand, HKP [6,7] retains the link connectivity, while it needs extra hash operations. Another approach to enhance NA resistance is unbalanced KP [15], which employs sensor nodes with different keyring sizes.

Because sensor nodes have a power limit constraint, it is a critical issue to save energy consumption in WSNs. This situation becomes more serious for a large-scale sensor network. One possible approach to reducing energy consumption is to deploy a multiple-sink WSN (MWSN) [20,21,22,23,24,25], for which most researchers adopt computation and topology techniques to improve connectivity between sensor nodes and sink nodes for energy reduction. In this paper, we study pairwise key predistribution in the MWSN environment to establish a short path collecting data via various sink nodes to improve energy efficiency in a large-scale WSN. The motivation of studying pairwise key predistribution in MWSNs will be further described in Section 3. Based on the new MWSN environment, where there are different clusters (groups), we formally define intragroup and intergroup conditions for our intragroup and intergroup key predistribution (I^2^KP). Three I^2^KP schemes are proposed, and each has its own advantages. The rest of this paper is organized as follows. Section 2 briefly reviews KP and HKP. In Section 3, the research motivation is described. Also, intragroup and intergroup conditions are defined. Three types of I^2^KP satisfying intragroup and intergroup conditions are proposed in Section 4. Comparisons and numerical simulations are given in Section 5 to show their pros and cons. A conclusion is drawn in Section 6.

## 2. Previous Works

### 2.1. Hash Chain

The one-way hash chain is an important cryptographic technology based on the hash function and can be used in many security applications. A hash function has the one-way property, which can undergo a collision attack and a preimage attack. Let the notation $h^{i}(x)=\overset{hash\;i\;times}{\overset{︷}{h(h(\cdots h(x)\cdots ))}}$ denote applying the hash function “*i*” times. We can create an *L*-length hash chain $\left(h^{0}(x),\;h^{1}(x),\;\ldots ,\;h^{L-1}(x)\right)$, where $h^{i}(x)=h(h^{i-1}(x))$ for 1 ≤ *i* ≤ (*L* − 1) and $h^{0}(x)=x$. Suppose the two hash indices are $i_{1}$ and $i_{2}$ ($i_{1}<i_{2}$); one can perform the *h*(·) function $\left(i_{2}-i_{1}\right)$ times to obtain $h^{i_{2}}(x)$ from $h^{i_{1}}(x)$ via $h^{i_{2}-i_{1}}(h^{i_{1}}(x))=h^{i_{2}}(x)$. Obviously, we cannot compute the hash chain backwards, i.e., $h^{i_{2}}(x)↛h^{i_{1}}(x)$.

### 2.2. KP and HKP

KP is a probabilistic scheme [5,10,11,15]. Each sensor is pre-loaded with m keys, where m is the key ring size. These keys are randomly selected from a key pool S with a size of |S|. The notions of various key predistribution schemes (HKP [6], HKP using a key-chain length *L* [7], *q*-composite KP [12,13], *q*-composite HKP (a hybrid of *q*-composite and HKP)) are briefly described as follows. *q*-composite KP establishes a secure link with at least *q* common keys between two neighboring nodes, and the *q*-composite KP with *q* = 1 is reduced as the conventional KP. Obviously, the large value of *q* more effectively resists NAs, but the link connectivity is reduced. Another way for improving NA resiliency is HKP. Each sensor node stores the hash key $h^{i}(x)$, where *i* is the node ID, unlike the KP that stores the raw key. HKP has the same link connectivity as KP, but it needs extra hash operations. This is because any two nodes (say node #*a* and node #*b*) storing $h^{i_{a}}(x)$ and $h^{i_{b}}(x)$, where $i_{a}<i_{b}$, may share a secret key $h^{i_{b}}(x)$. For this case, node #*b* uses its hashed key $h^{i_{b}}(x)$, while node #*a* obtains $h^{i_{b}}(x)$ from its hashed key $h^{i_{a}}(x)$ by using $h^{\left(i_{b}-i_{a}\right)}(h^{i_{a}}(k))=h^{i_{b}}(k)$. Meantime, HKP can improve NA resiliency. Suppose a captured node #*c* has a hashed key $h^{i_{c}}(x)$ in its key ring and that the hash index $i_{c}$ is greater than $i_{b}$. If node #*c* is compromised, the hashed key $h^{i_{c}}(k)$ cannot be used to eavesdrop in the link between nodes #*a* and #*b*c because attackers cannot compute $h^{i_{b}}(k)$ backward from $h^{i_{c}}(k)$. To further enhance NA resiliency, HKP using a parameter key-chain length *L* is introduced. Namely, we can store the hashed key in $h^{i}(k)$, where 0 ≤ *i* ≤ (*L* − 1), for a sensor node according to its node ID (mod *L*). When the key-chain length *L* is used, the number of hash operations is limited to *L*, thereby reducing the computational complexity.

**Figure 1:**
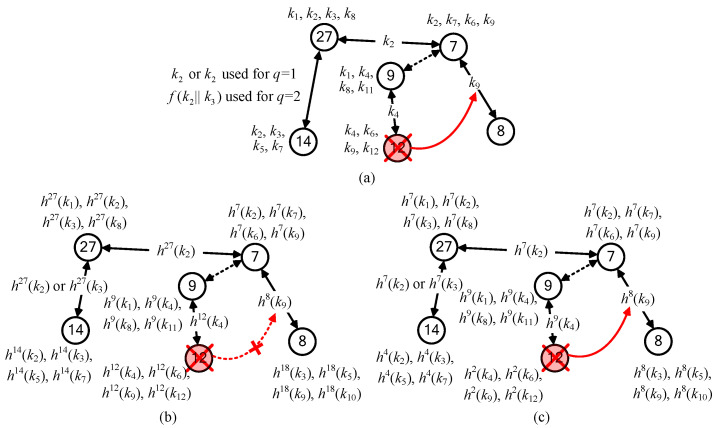
Various KP schemes: (**a**) 1-composite KP and 2-composite KP; (**b**) 1-composite HKP; (**c**) 1-composite HCKP using key-chain length *L* = 10.

Next, we use an example including six sensor nodes with IDs #7, #8, #9, #12, #14, and #27 to briefly describe *q*-composite KP and HKP. As illustrated in Figure 1, the key ring size is *m* = 4. Figure 1a is single-composite KP (*q* = 1). There are four links with *q* ≥ 1 for any two neighbor nodes: (#7↔#8), (#9↔#12), (#7↔#27), and (#14↔#27). For example, nodes #14 and #27 have the same two keys (*k*_2_ and *k*_3_) in their key rings. Since *q* = 1, these two nodes can use any one key (*k*_2_ or *k*_3_) to establish a secure link. Consider the case *q* = 2 (dual-composite KP). Two nodes need *q*′ ≥ 2 keys (say $k_{1},\;k_{2},\;\ldots ,\;k_{q^{\prime }}$) to establish a secure link. For dual-composite KP, only one link (#14↔#27) with a hybrid of $k_{2}$ and $k_{3}$ (for example, using a hashed key $f(k_{2}||k_{3})$) can be established. The large *q* enhances NA resiliency because attackers need more captured keys to obtain the hashed keys. However, this is also the reason that the connectivity is reduced, because the link is established by *q* keys. For the single-composite scheme in Figure 1a, when node #12 is captured, attackers having $k_{9}$ can compromise the link (#7↔#8). But, for the case *q* = 2, attackers cannot compromise this link because this link (#7↔#8) cannot be established.

*q*-composite HKP is *q*-composite KP but storing the hashed keys in a key ring. Figure 1b is single-composite HKP. Similarly to single-composite KP in Figure 1a, all the secure links for (#7↔#8), (#9↔#12), (#7↔#27), and (#14↔#27) can be established by using hashed keys instead of raw keys. For example, the link (#7↔#27) in Figure 1a uses the raw key $k_{2}$, but the link (#7↔#27) in Figure 1b uses the hashed key $h^{27}(k_{2})$. It is observed that the connectivity of single-composite HKP is not reduced, but node #7 needs extra 20 hash operations to obtain $h^{27}(k_{2})$ from $h^{7}(k_{2})$. The NA resiliency of single-composite HKP is enhanced. For example, in Figure 1b, the hashed key $h^{12}(k_{9})$ in the captured node #12 cannot be used to compromise the link (#7↔#8) using $h^{8}(k_{9})$ since attackers cannot backward- compute the hash chain. As described above, *q*-composite HKP needs hash operations between two nodes #*i* and #*j* to share a common key. Thus, the large scale of the WSN results in heavy computation overhead. To reduce hash operations, a parameter key-chain length *L* is introduced. The hashed keys $h^{i}(k)$ and $h^{j}(k)$ in nodes #*i* and #*j* are replaced by $h^{i(\mathrm{mod}\;L)}(k)$ and $h^{j(\mathrm{mod}\;L)}(k)$. Finally, the number of required hash operations |i(mod L)−j(mod L)| for establishing a pairwise key is reduced and bounded by *L*. Single-composite HCKP using *L* = 10 is shown in Figure 1c, in which both nodes #7 and #27 use the hashed key $h^{7}(k_{2})$ to establish a secure link. Compared with Figure 1b, using *L* = 10 does not need extra hash operations for the link (#7↔#27). Although using *L* reduces hash operations, its resiliency against NAs is also degraded. We use the following case to show this reduction in resiliency. As shown in Figure 1c, the attacker can capture node #12 with $h^{2}(k_{9})$ to obtain $h^{8}(k_{9})$ and then compromise the link (#7↔#8) using the hashed key $h^{8}(k_{9})$. However, in Figure 1b, the attacker captures node #12 but cannot compromise the link (#7↔#8) because he cannot obtain $h^{8}(k_{9})$ from $h^{12}(k_{9})$.

## 3. Motivation

Due to the power limit constraint of sensors, it is a critical issue to save energy consumption in WSNs, especially in a large-scale sensor network. One possible solution for reducing energy consumption and, in the meantime, maintaining performance with the increment in sensor nodes is to deploy a multiple-sink WSN (MWSN) [20,21,22,23,24,25]. The above implies that the MWSN approach could deal with concerns about “energy consumption” and “increment of sensor nodes” in WSNs. This motivates us to study secure communications in MWSNs.

**Figure 2:**
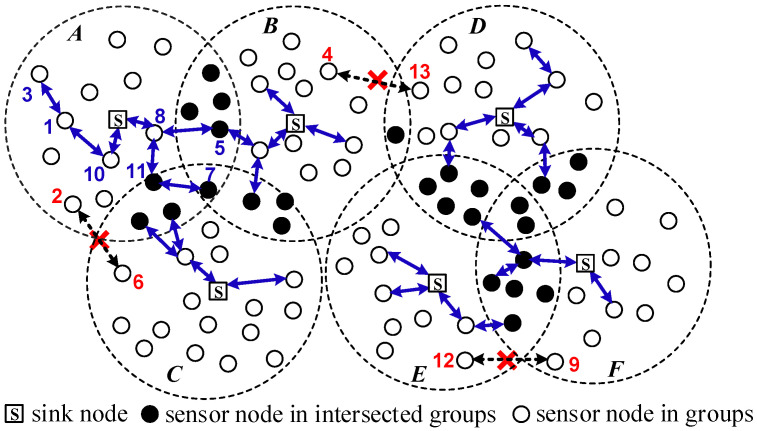
Pairwise key predistribution in MWSN.

An MWSN includes a number of clusters (groups) with a sink node for each group. As illustrated in Figure 2, there are six groups *A*~*F* in this MWSN.

The sensor nodes in a group can gather data and send them back to the backend database via a sink node in its group. In this paper, we study KP in an MWSN environment. We first consider an intragroup case with all the nodes in a group (say group *A*). Suppose any two neighbor nodes in the following links, (#3↔1↔#10↔S), (#5↔#8↔S), and (#7↔#11↔#8↔S), have a common key; then, nodes #3, #5, and #7 can gather data and send them back to the sink node through these secure links, respectively. It is observed that nodes #5 and #11 are in two intersecting groups (*A* and *B*) and (*A* and *C*), and the node #7 is in three intersecting groups (*A*, *B,* and *C*). The nodes in intersecting groups can connect with nodes in different groups. For example, node #5 can send data back through the sink node in group *B*. Next, we discuss the intergroup case. The nodes in different groups which are not located in intersecting areas have no same key in their key rings. Thus, they cannot establish a secure link. For example, the following could not establish secure links: node #2 in group *A* and node #6 in group *B* (#2
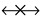
#6), node #4 in group *B* and node #13 in group *D* (#4
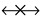
#13), and node #12 in group *E* and node #9 in group *F* (#12
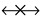
#9). The reason we do not let the nodes in two groups establish secure links is to control the stream flow diversion from data collection for saving on energy consumption.

Based on the above observations, we formally define intragroup and intergroup conditions when dealing with I^2^KP in an MWSN environment.

(1) Intragroup condition: The nodes in each group with a common sink node have a pairwise key predistribution ability like in KP. The nodes located in more intersecting groups have the highest probability to establish secure links with other neighboring nodes in the whole MWSN.
(2) Intergroup condition: The nodes in different groups are disjointed pairwise for the key ring, and, thus, they do not have the same keys to establish secure links.

Condition (1) lets the node pairwisely share a common key between two neighbor nodes to gather data and send them back via the sink node within one group. If the nodes are in intersecting groups, the secure links and the sink nodes may be more than one. This condition assures I^2^KP has the capability of using multiple sink nodes to gather data. For this case, the nodes in intersecting groups could have different path selections (it has higher connectivity than others that are not in intersecting groups) to pass collected data via various sink nodes, and we can use path switching to improve energy efficiency. Regarding condition (2), node #2 in group *A* and node #6 in group *C* are in different groups (see Figure 2). Thus, nodes #2 and #6 are not in intersected groups and are disjointed pairwise for the key ring. Generally, the nodes in two pairwise-disjointed areas for the key ring are some distance away. If they cannot establish a link, we have a short path selection to improve energy efficiency. The above motivates us to study I^2^KP for large-scale MWSNs.

## 4. The Proposed I^2^KP

Three types of I^2^KP are designed. The first is I^2^KP using different key pools (I^2^KPDP). I^2^KPDP is a trivial way to use conventional pairwise key predistribution for each group. However, the key ring size will be expanded for the nodes in intersecting groups. To reduce the key ring size of the sensor node, I^2^KP using the same key pool for all groups (I^2^KPSP) is proposed. I^2^KPDP and I^2^KPSP cannot resist a location-based NA (LNA). Attackers may know which nodes belong to which group from their locations. If an attacker has knowledge of the geometric locations of sensor nodes, he may intentionally capture the nodes in intersected groups to aggravate the NA. Both I^2^KPDP and I^2^KPSP suffer from this LNA. We design I^2^KP using a hash chain (I^2^KPHC) to effectively tackle LNAs.

### 4.1. The Proposed I^2^KPDP

**Figure 3:**
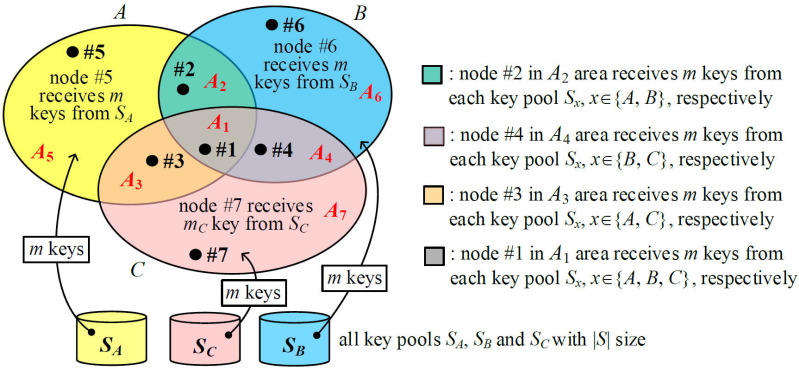
The proposed I^2^KPDP.

We propose I^2^KPDP by using different a key pool for each group. Figure 3 illustrates an example of three groups, *A, B*, and *C*. Suppose that the number of all possible key rings of size $m_{x}$ selected from a key pool $S_{x}$ of size $\left|S_{x}\right|$ with no identical keys in the key rings, where *x*∈{*A*, *B*, *C*}, i.e., $S_{A}\cap S_{B}=\phi$, $S_{A}\cap S_{C}=\phi$, and $S_{B}\cap S_{C}=\phi .$ The key selection rules for I^2^KPDP are described below. For a node in group *x*, $m_{x}$ keys are randomly selected from the key pool $S_{x}$. Thus, if the node is in the group (x∩y), there are a total of $\left(m_{x}+m_{y}\right)$ keys in its key ring. Obviously, the node in the group (x∩y∩z) will have $\left(m_{x}+m_{y}+m_{z}\right)$ keys. For simplicity, we use $m=m_{x}=m_{y}=m_{z}$ and $\left|S|=|S_{x}|=|S_{y}|=|S_{z}\right|$ to describe I^2^KPDP. Seven disjointed areas *A_i_*, 1 ≤ *i* ≤ 7, with a size of $|A_{i}|=n_{i}$ in Figure 3 are represented in Equation (1). Finally, nodes #5, #6, and #7 in areas *A*_5_, *A*_6_, and *A*_7_ have a key ring size of *m*. Nodes #2, #3, and #4 in areas *A*_2_, *A*_3_, and *A*_4_ have a key ring size of 2*m*, while node #1 in the *A*_1_ area has a key ring size of 3*m*.


(1) $$\left\{\begin{array}{l} A_{1}=(A\cap B\cap C);\;A_{2}=(A\cap B)-(A\cap B\cap C);\;A_{3}=(A\cap C)-(A\cap B\cap C)\\ A_{4}=(B\cap C)-(A\cap B\cap C);\;A_{5}=A-(A\cap B)-(A\cap C)+(A\cap B\cap C)\\ A_{6}=B-(A\cap B)-(B\cap C)+(A\cap B\cap C);\;A_{7}=C-(A\cap C)-(B\cap C)+(A\cap B\cap C) \end{array}\right.$$

Via [6,7], we could derive Equation (2) expressing the probability that any two nodes in the network group *x* ∈{*A*, *B*, *C*} share exactly *l* independent keys from the key pool $S_{x}$, where $\left|S_{x}|=|S\right|$.

(2) $$P_{Share}^{\left(x\right)}(l)=\left(\begin{array}{c} \left|S\right|\\ l \end{array}\right)\left(\begin{array}{c} |S|-l\\ 2(m-l) \end{array}\right)\left(\begin{array}{c} 2(m-l)\\ m-l \end{array}\right)/{\left(\begin{array}{c} \left|S\right|\\ m \end{array}\right)}^{2}$$

Let $P_{Share}^{\left(x\cap y\right)}(l)$ be the probability that any two nodes in (x∩y) share exactly *l* independent keys and $P_{Share}^{\left(x\cap y\cap z\right)}(l)$ be the probability that any two nodes in (x∩y∩z) share exactly *l* independent keys. Based on Equation (2), $P_{Share}^{\left(x\cap y\right)}(l)$ and $P_{Share}^{\left(x\cap y\cap z\right)}(l)$ are derived in Equation (3), where $P_{Share}^{\left(x\right)}(l)=P_{Share}^{\left(y\right)}(l)=P_{Share}^{\left(z\right)}(l)$.

(3) $$\left\{\begin{array}{l} P_{Share}^{\left(x\cap y\right)}(l)=\sum_{0\leq l_{1},\;l_{2}\leq l\;\mathrm{and}\;l_{1}+l_{2}=l}\left(P_{Share}^{\left(x\right)}(l_{1})+P_{Share}^{\left(y\right)}(l_{2})\right)\\ P_{Share}^{\left(x\cap y\cap z\right)}(l)=\sum_{0\leq l_{1},\;l_{2},\;l_{3}\leq l\;\mathrm{and}\;l_{1}+l_{2}+l_{3}=l}\left(P_{Share}^{\left(x\right)}(l_{1})+P_{Share}^{\left(y\right)}(l_{2})+P_{Share}^{\left(z\right)}(l_{3})\right) \end{array}\right.$$

**Figure 4:**
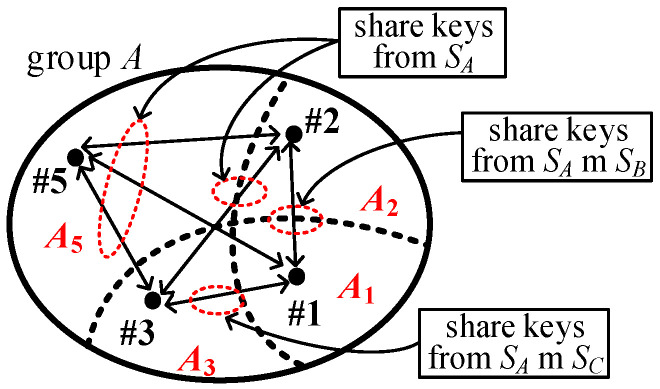
The keys used between subgroups in group *A* for I^2^KPDP.

Obviously, any two nodes in *x*, (x∩y), and (x∩y∩z) may share the common keys from the key pools $S_{x}$, $\left(S_{x}\cup S_{y}\right)$, and $\left(S_{x}\cup S_{y}\cup S_{z}\right)$, respectively. The following shows how any two nodes in the whole group G=A∪B∪C share the common keys. However, if one node is in *x* and the other node is in (x∩y), they could only use the common key pool $S_{x}$. Figure 4 shows the cases between subgroups in group *A*.

The links (#5↔#1), (#5↔#2), (#5↔#3), and (#2↔#3) use the keys from $S_{A}$, while the links (#1↔#2) and (#1↔#3) use the keys from $\left(S_{A}\cup S_{B}\right)$ and $\left(S_{A}\cup S_{C}\right)$, respectively. Because we use the condition $m=m_{x}=m_{y}=m_{z}$ and $\left|S|=|S_{x}|=|S_{y}|=|S_{z}\right|$, we have $P_{Share}^{\left(x\right)}(l)=P_{Share}^{\left(y\right)}(l)$ $=P_{Share}^{\left(z\right)}(l)$ and $P_{Share}^{\left(x\cap y\right)}(l)=P_{Share}^{\left(y\cap z\right)}(l)=P_{Share}^{\left(x\cap z\right)}(l)$. To more simply denote the probabilities, we use $P_{Share}^{\left(1\right)}(l)$, $P_{Share}^{\left(2\right)}(l)$, and $P_{Share}^{\left(3\right)}(l)$ to represent the probabilities for single-intersected, double-intersected and triple-intersected groups, respectively (note: $P_{Share}^{\left(1\right)}(l)$ is the same as that in the conventional KP). The probabilities $P_{Share}^{\left(A\right)}(l)$, $P_{Share}^{\left(B\right)}(l)$, and $P_{Share}^{\left(C\right)}(l)$ using the keys from $S_{A}$, $S_{B}$, and $S_{C}$, respectively, share exactly *l* independent keys for I^2^KPDP as derived in Lemma 1.

**Lemma** **1.** *The intragroup probabilities* $P_{Share}^{\left(A\right)}(l)$*, *$P_{Share}^{\left(B\right)}(l)$*, and *$P_{Share}^{\left(C\right)}(l)$ *for I^2^KPDP, as shown in Figure 2, are derived in Equations (4), (5), and (6), respectively.*

**Proof.**  As illustrated in Figure 3, we consider that one node *i* in $A_{1}$, $A_{2}$, $A_{3}$, and $A_{5}$ (note: $A=A_{1}\cup A_{2}\cup$ $A_{3}\cup A_{5}$), respectively, and one node *j* in *A* to communicate with each other (see Figure 4). All the possible choices of nodes *i* and *j* in *A* are $|A{|}^{2}=n^{2}$. Finally, the average probability $P_{Share}^{\left(A\right)}(l)$ is derived as follows.

(4) $$\left\{\begin{array}{l} P_{Share}^{\left(A\right)}(l)=\left(\begin{array}{c} \overset{\mathrm{node}\;i\;\mathrm{in}\;A_{1}\;\mathrm{node}\;j\;\mathrm{in}\;A_{1}}{\overset{︷}{\sum_{i\in A_{1}}\sum_{j\in A_{1}}P_{Share}^{\left(3\right)}(l)}}+\overset{\mathrm{node}\;i\;\mathrm{in}\;A_{1}\;\mathrm{node}\;j\;\mathrm{in}\;\text{\{}A_{2},\;A_{3}\}}{\overset{︷}{\sum_{i\in A_{1}}\sum_{j\in \text{\{}A_{2},\;A_{3}\}}P_{Share}^{\left(2\right)}(l)}}+\overset{\mathrm{node}\;i\;\mathrm{in}\;A_{1}\;\mathrm{node}\;j\;\mathrm{in}\;A_{5}}{\overset{︷}{\sum_{i\in A_{1}}\sum_{j\in A_{5}}P_{Share}^{\left(1\right)}(l)}}\\ +\overset{\mathrm{node}\;i\;\mathrm{in}\;A_{2}\;\mathrm{node}\;j\;\mathrm{in}\;(A_{1}\cup A_{2})}{\overset{︷}{\sum_{i\in A_{2}}\sum_{j\in (A_{1}\cup A_{2})}P_{Share}^{\left(2\right)}(l)}}+\overset{\mathrm{node}\;i\;\mathrm{in}\;A_{2}\;\mathrm{node}\;j\;\mathrm{in}\;A-(A_{1}\cup A_{2})}{\overset{︷}{\sum_{i\in A_{2}}\sum_{j\in \{A-(A_{1}\cup A_{2})\}}P_{Share}^{\left(1\right)}(l)}}+\\ \overset{\mathrm{node}\;i\;\mathrm{in}\;A_{3}\;\mathrm{node}\;j\;\mathrm{in}\;(A_{1}\cup A_{3})}{\overset{︷}{\sum_{i\in A_{3}}\sum_{j\in (A_{1}\cup A_{3})}P_{Share}^{\left(2\right)}(l)}}+\overset{\mathrm{node}\;i\;\mathrm{in}\;A_{3}\;\mathrm{node}\;j\;\mathrm{in}\;A-(A_{1}\cup A_{3})}{\overset{︷}{\sum_{i\in A_{3}}\sum_{j\in \{A-(A_{1}\cup A_{3})\}}P_{Share}^{\left(1\right)}(l)}}+\overset{\mathrm{node}\;i\;\mathrm{in}\;A_{5}\;\mathrm{node}\;j\;\mathrm{in}\;A}{\overset{︷}{\sum_{i\in A_{5}}\sum_{j\in A}P_{Share}^{\left(1\right)}(l)}} \end{array}\right)/|A{|}^{2}\\ =\left(\begin{array}{c} \left(n_{5}\times (2n-n_{5})+2n_{2}n_{3}\right)\cdot P_{Share}^{\left(1\right)}(l)+\left(n_{2}^{2}+n_{3}^{2}\right.\\ \left.+2n_{1}(n_{2}+n_{3})\right)\cdot P_{Share}^{\left(2\right)}(l)+n_{1}^{2}\cdot P_{Share}^{\left(3\right)}(l) \end{array}\right)/n^{2} \end{array}\right.$$

By the same argument, $P_{Share}^{\left(B\right)}(l)$ and $P_{Share}^{\left(C\right)}(l)$ are derived in Equations (5) and (6).

(5) $$\left\{P_{Share}^{\left(B\right)}(l)=\left(\begin{array}{c} \left(n_{6}\times (2n-n_{6})+2n_{2}n_{4}\right)\cdot P_{Share}^{\left(1\right)}(l)+\left(n_{2}^{2}+n_{4}^{2}\right.\\ \left.+2n_{1}(n_{2}+n_{4})\right)\cdot P_{Share}^{\left(2\right)}(l)+n_{1}^{2}\cdot P_{Share}^{\left(3\right)}(l) \end{array}\right)/n^{2}\right.$$

(6) $$\left\{P_{Share}^{\left(C\right)}(l)=\left(\begin{array}{c} \left(n_{7}\times (2n-n_{7})+2n_{3}n_{4}\right)\cdot P_{Share}^{\left(1\right)}(l)+\left(n_{3}^{2}+n_{4}^{2}\right.\\ \left.+2n_{1}(n_{3}+n_{4})\right)\cdot P_{Share}^{\left(2\right)}(l)+n_{1}^{2}\cdot P_{Share}^{\left(3\right)}(l) \end{array}\right)/n^{2}\right.$$

Based on $P_{Share}^{\left(x\right)}(l)$, the probability that two given intragroup nodes establish a secure link is $P_{Link-Est}^{\left(x\right)}=\sum_{l=q}^{m}P_{Share}^{\left(x\right)}(l)$, where *x*, *y*, *z*∈{*A*, *B*, *C*}. Next, we prove that our I^2^KPDP satisfies the intragroup and intergroup conditions. □

**Theorem** **1.** *The proposed q-composite I^2^KPDP in Figure 3 satisfies intragroup and intergroup conditions.*

**Proof.**  We first prove that *q*-composite I^2^KPDP satisfies the intragroup condition. In each group, we have the probability $P_{Link-Est}^{\left(A\right)}$, $P_{Link-Est}^{\left(B\right)}$, and $P_{Link-Est}^{\left(C\right)}$ to establish a secure link. The link connectivity is the same as the connection like in KP. Since the nodes in more intersecting groups have a higher probability of establishing a link, we consider the node in single-intersected (the nodes in *A*_5_, *A*_6_, and *A*_7_), double-intersected (the nodes in *A*_2_, *A*_3_, and *A*_4_), and triple-intersected (the nodes in *A*_1_) groups. Let $G_{Link-Area}^{\left(A_{i}\to G\right)}$, where $G=A\cup B\cup C=\cup_{i=1}^{7}A_{i}$, be the set of subgroups that the nodes in $A_{i}$ could establish a secure link with the nodes in this set. Obviously, Equation (7) implies that the nodes in more intersected groups can establish a link with more nodes in the whole group *G*.

(7) $$\begin{array}{l} \left\{\begin{array}{l} G_{Link-Est}^{\left(A_{5}\to G\right)}=\{A_{1},\;A_{2},\;A_{3},\;A_{5}\}\Rightarrow \left|G_{Link-Est}^{\left(A_{5}\to G\right)}\right|=\left|A_{1}+A_{2}+A_{3}+A_{5}\right|\\ G_{Link-Est}^{\left(A_{2}\to G\right)}=\{A_{1},\;A_{2},\;A_{3},\;A_{4},\;A_{5},\;A_{6}\}\Rightarrow \left|G_{Link-Est}^{\left(A_{2}\to G\right)}\right|=\left|A_{1}+A_{2}+A_{3}+A_{4}+A_{5}+A_{6}\right|\\ G_{Link-Est}^{\left(A_{1}\to G\right)}=\{A_{1},\;A_{2},\;A_{3},\;A_{4},\;A_{5},\;A_{6},\;A_{7}\}\Rightarrow \left|G_{Link-Est}^{\left(A_{1}\to G\right)}\right|=\left|A_{1}+A_{2}+A_{3}+A_{4}+A_{5}+A_{6}+A_{7}\right| \end{array}\right.\\ \Rightarrow \left|G_{Link-Est}^{\left(A_{1}\to G\right)}\right|>\left|G_{Link-Est}^{\left(A_{2}\to G\right)}\right|>\left|G_{Link-Est}^{\left(A_{5}\to G\right)}\right| \end{array}$$

Next, we prove the intergroup condition. The nodes in the two groups *A* and *B* obtain the keys from key pools $S_{A}$ and $S_{B}$, respectively, where $S_{A}\cap S_{B}=\phi$. Thus, the nodes in group *A* and the nodes in group *B* cannot establish secure links except in the intersected group (A∩B). The above implies that the proposed I^2^KPDP satisfies intragroup and intergroup conditions. □

The resiliency against NAs for our I^2^KPDP is described as follows. I^2^KPDP dealing with one group is reduced as in conventional KP. We first show NA resiliency for conventional KP and then give a formal analysis of our I^2^KPDP. Suppose that *k* nodes are captured in a random fashion, and the stored keys are compromised. Because each node contains *m* keys, the probability that a given key is uncompromised is ${\left(\begin{array}{c} 1-m \end{array}/\left|S\right|\right)}^{k}$. On the contrary, the probability that a given key has been known is $1-{\left(\begin{array}{c} 1-m \end{array}/\left|S\right|\right)}^{k}$. If the key of a link between two nodes is the hybrid of *l* shared keys, the probability of that link being compromised is ${\left(1-{\left(1-\begin{array}{c} m \end{array}/\left|S\right|\right)}^{k}\right)}^{l}$. Then, the probability that any secure link between two uncompromised nodes is compromised when *k* nodes are captured in group *x* is calculated in Equation (8), which is the same as in KP [6,7].


(8) $$P_{Link-Com}^{\left(x\right)}=\sum_{l=q}^{m}{\left(1-{\left(1-\begin{array}{c} m \end{array}/\left|S\right|\right)}^{k}\right)}^{l}\cdot P_{Share}^{\left(x\right)}(l)/\sum_{l=q}^{m}P_{Share}^{\left(x\right)}(l)$$

For the proposed I^2^KPDP, the nodes in different areas have a different number of keys in their key rings. Thus, we should give different analyses for the probability that any secure link between two uncompromised nodes in G=(A∪B∪C) is compromised when *k* nodes are captured. For *k* captured nodes, it is not reasonable to consider all *k* captured nodes are randomly distributed in the large-scale MWSN *G* with many groups (say 200 groups). The case “*k* captured nodes” in such a large-scale *G* does not work for an NA, because there is a very low probability that the *k* captured nodes are selected from the same key pool. An NA attack does not work in this case. The following scenario about an NA in I^2^KPDP is more possible and reasonable. The attacker could know the nodes belonging to which group, but he does not know whether the nodes are located in intersected groups. As we know, the probability $P_{Link-Com}^{\left(x\to x\right)}$ of I^2^KPDP is the same as $P_{Link-Com}^{\left(x\right)}$ in conventional KP. Therefore, we discuss the probabilities $P_{Link-Com}^{\left(x\to G\right)}$, where *x*∈{*A*, *B*, *C*}, for I^2^KPDP that any secure link between two uncompromised nodes is compromised in the whole group *G* when *k* nodes are captured in a random fashion in group *x*. All the probabilities $P_{Link-Com}^{\left(A\to G\right)}$, $P_{Link-Com}^{\left(B\to G\right)}$, and $P_{Link-Com}^{\left(C\to G\right)}$ are derived in Theorem 2. To simply represent the probabilities $P_{Link-Com}^{\left(A\to G\right)}$, $P_{Link-Com}^{\left(B\to G\right)}$, and $P_{Link-Com}^{\left(C\to G\right)}$, assume that the intersecting group does not have many sensors (note: this is a reasonable assumption). Suppose $n_{1}=n_{2}=n_{3}=n_{4}=$ n/8, and thus we have $n_{5}=n_{6}=n_{7}=5n/8$. By Equations (4)–(6), the values of $P_{Share}^{\left(A\right)}(l)=P_{Share}^{\left(B\right)}(l)=P_{Share}^{\left(C\right)}(l)$ are $\left(57P_{Share}^{\left(1\right)}+6P_{Share}^{\left(2\right)}+P_{Share}^{\left(3\right)}\right)/64$. The result implies that we can use $P_{Share}^{\left(x\right)}(l)\approx P_{Share}^{\left(1\right)}$ to simply derive $P_{Link-Com}^{\left(x\to G\right)}$, where *x*∈{*A*, *B*, *C*}.

**Theorem** **2.** *In q-composite I^2^KPDP, when k nodes are captured randomly in groups A, B, and C, respectively, the probabilities* $P_{Link-Com}^{\left(A\to G\right)}$*, $P_{Link-Com}^{\left(B\to G\right)}$, and $P_{Link-Com}^{\left(C\to G\right)}$of a link being compromised are shown in Equations (9)–(11).*

(9) $$P_{Link-Com}^{\left(A\to G\right)}\approx \left(\frac{C_{k}^{n}+C_{k}^{n_{1}+n_{2}}+C_{k}^{n_{1}+n_{3}}}{3C_{k}^{n}}\right)\cdot \left(\sum_{l=q}^{m}{\left(1-{\left(1-\frac{m}{\left|S\right|}\right)}^{k}\right)}^{l}\cdot \frac{P_{Share}^{\left(1\right)}(l)}{\sum_{l=q}^{m}P_{Share}^{\left(1\right)}(l)}\right)$$

(10) $$P_{Link-Com}^{\left(B\to G\right)}\approx \left(\frac{C_{k}^{n}+C_{k}^{n_{1}+n_{2}}+C_{k}^{n_{1}+n_{4}}}{3C_{k}^{n}}\right)\cdot \left(\sum_{l=q}^{m}{\left(1-{\left(1-\frac{m}{\left|S\right|}\right)}^{k}\right)}^{l}\cdot \frac{P_{Share}^{\left(1\right)}(l)}{\sum_{l=q}^{m}P_{Share}^{\left(1\right)}(l)}\right)$$

(11) $$P_{Link-Com}^{\left(C\to G\right)}\approx \left(\frac{C_{k}^{n}+C_{k}^{n_{1}+n_{3}}+C_{k}^{n_{1}+n_{4}}}{3C_{k}^{n}}\right)\cdot \left(\sum_{l=q}^{m}{\left(1-{\left(1-\frac{m}{\left|S\right|}\right)}^{k}\right)}^{l}\cdot \frac{P_{Share}^{\left(1\right)}(l)}{\sum_{l=q}^{m}P_{Share}^{\left(1\right)}(l)}\right)$$

**Proof.**  Suppose that *k* captured nodes are selected in a random fashion in group *A*. There are a total of $C_{k}^{n}$ ways to select *k* captured nodes. As illustrated in Figure 3, we consider four cases: (i) all *k* captured nodes are in *A*_1_, and there are $C_{k}^{n_{1}}$ types to select *k* captured nodes; (ii) all *k* captured nodes are in A∩B (i.e., $A_{1}\cup A_{2}$), excluding case (i), and there are $C_{k}^{n_{1}+n_{2}}-C_{k}^{n_{1}}$ types to select *k* captured nodes; (iii) all *k* captured nodes are in A∩C (i.e., $A_{1}\cup A_{3}$), excluding case (i), and there are $C_{k}^{n_{1}+n_{3}}-C_{k}^{n_{1}}$ types to select *k* captured nodes; (iv) all *k* captured nodes are in group *A*, excluding cases (i), (ii), and (iii), and there are $C_{k}^{n}-C_{k}^{n_{1}+n_{2}}-C_{k}^{n_{1}+n_{3}}+C_{k}^{n_{1}}$ types to select *k* captured nodes. The total number of these cases is $C_{k}^{n}$. For case (i), the captured keys from *S_A_*, *S_B_*, and *S_C_* can be used to compromise the links in groups *A*, *B,* and *C*. By the same argument, when *k* nodes are captured for cases (ii) and (iii), the information of captured nodes can be used for compromising the links in *A* and *B* as well as *A* and *C*, respectively. The case (iv) is to compromise the links in group *A* excluding cases (i), (ii), and (iii). By the approximation $P_{Share}^{\left(x\right)}(l)\approx P_{Share}^{\left(1\right)}(l)$ and the derivation of $P_{Link-Com}^{\left(x\right)}$ in Equation (8), the probability $P_{Link-Com}^{\left(A\to G\right)}$ is approximately derived in Equation (12). This probability is divided by three because the size of the whole set *G* is about three times the size of group *A*, i.e., |G|≈3×|A|. By the same argument, we could derive $P_{Link-Com}^{\left(B\to G\right)}$ and $P_{Link-Com}^{\left(C\to G\right)}$ in Equations (10) and (11).

(12) $$\left\{\begin{array}{l} P_{Link-Com}^{\left(A\to G\right)}\approx \frac{1}{3}\cdot \{\overset{\mathrm{case}\;(i)\text{:}\;\mathrm{compromise}\;\mathrm{the}\;\mathrm{links}\;\mathrm{in}\;3\;\mathrm{groups}\;A,\;B\;\mathrm{and}\;C}{\overset{︷}{\frac{C_{k}^{n_{1}}}{C_{k}^{n}}\cdot 3\left(\sum_{l=q}^{m}{\left(1-{\left(1-\frac{m}{\left|S\right|}\right)}^{k}\right)}^{l}\cdot \frac{P_{Share}^{\left(1\right)}(l)}{\sum_{l=q}^{m}P_{Share}^{\left(1\right)}(l)}\right)}}+\overset{\mathrm{case}\;(\mathrm{ii})\text{:}\;\mathrm{compromise}\;\mathrm{the}\;\mathrm{links}\;\mathrm{in}\;2\;\mathrm{groups}\;A\;\mathrm{and}\;B}{\overset{︷}{\frac{C_{k}^{n_{1}+n_{2}}-C_{k}^{n_{1}}}{C_{k}^{n}}\cdot 2\left(\sum_{l=q}^{m}{\left(1-{\left(1-\frac{m}{\left|S\right|}\right)}^{k}\right)}^{l}\cdot \frac{P_{Share}^{\left(1\right)}(l)}{\sum_{l=q}^{m}P_{Share}^{\left(1\right)}(l)}\right)}}\\ +\overset{\mathrm{case}\;(\mathrm{iii})\text{:}\;\mathrm{compromise}\;\mathrm{the}\;\mathrm{links}\;\mathrm{in}\;2\;\mathrm{groups}\;A\;\mathrm{and}\;C}{\overset{︷}{\frac{C_{k}^{n_{1}+n_{3}}-C_{k}^{n_{1}}}{C_{k}^{n}}\cdot 2\left(\sum_{l=q}^{m}{\left(1-{\left(1-\frac{m}{\left|S\right|}\right)}^{k}\right)}^{l}\cdot \frac{P_{Share}^{\left(1\right)}(l)}{\sum_{l=q}^{m}P_{Share}^{\left(1\right)}(l)}\right)}}+\overset{\mathrm{case}\;(\mathrm{iv})\text{:}\;\mathrm{compromise}\;\mathrm{the}\;\mathrm{links}\;\mathrm{in}\;\mathrm{group}\;A}{\overset{︷}{\frac{C_{k}^{n}-C_{k}^{n_{1}+n_{2}}-C_{k}^{n_{1}+n_{3}}+C_{k}^{n_{1}}}{C_{k}^{n}}\cdot \left(\sum_{l=q}^{m}{\left(1-{\left(1-\frac{m}{\left|S\right|}\right)}^{k}\right)}^{l}\right.}}\\ \left.\cdot \frac{P_{Share}^{\left(1\right)}(l)}{\sum_{l=q}^{m}P_{Share}^{\left(1\right)}(l)}\right))=\left(\frac{C_{k}^{n}+C_{k}^{n_{1}+n_{2}}+C_{k}^{n_{1}+n_{3}}}{3C_{k}^{n}}\right)\cdot \left(\sum_{l=q}^{m}{\left(1-{\left(1-\frac{m}{\left|S\right|}\right)}^{k}\right)}^{l}\cdot \frac{P_{Share}^{\left(1\right)}(l)}{\sum_{l=q}^{m}P_{Share}^{\left(1\right)}(l)}\right) \end{array}\right.$$

I^2^KPDP that adopts different key pools for different groups increases the key ring size. Sensor nodes in the intersected groups (x∩y) and (x∩y∩z) must store 2*m* and 3*m* keys in the key ring, respectively. To reduce the storage sizes of sensors in intersected groups, we use the same key pool for all groups to design I^2^KPSP. □

### 4.2. The Proposed I^2^KPSP

**Figure 5:**
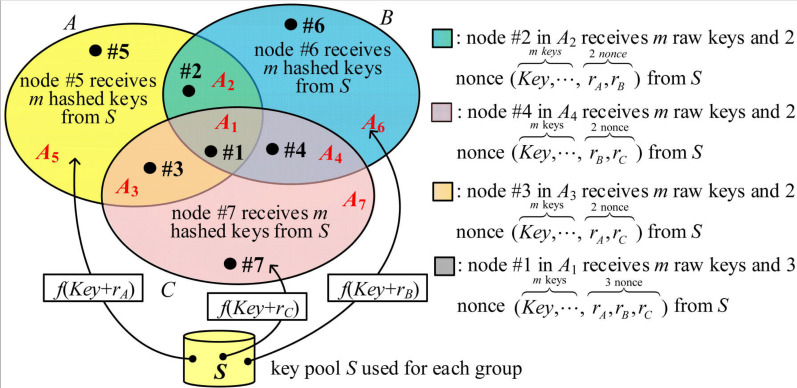
The proposed I^2^KPSP.

The same key pool is used for all groups to reduce the key ring size. We also use three groups, *A*, *B*, and *C*, to describe I^2^KPSP (see Figure 5). For the key selection rules in I^2^KPSP, a nonce is randomly selected for a group. As illustrated in Figure 5, three nonces, *r_A_*, *r_B_*, and *r_C_*, and one hash function f(⋅) are used in I^2^KPSP. For the nodes in (x−(x∩y)−(x∩z)), where *x*, *y*, *z*∈{*A*, *B*, *C*}, if *m* keys (say $K_{i},\;1\leq i\leq m$) are randomly selected from key pool *S*, then *m* hashed keys $\left(f(K_{i}+r_{x}),\;1\leq i\leq m\right)$ are delivered to this node. For the nodes in (x∩y), (*m* + 2) values including *m* raw keys and two nonce $\left(\overset{m\;\mathrm{keys}}{\overset{︷}{K_{i},\;1\leq i\leq m}},\overset{2\;\mathrm{nonce}}{\overset{︷}{r_{x},r_{y}}}\right)$ are given to the nodes, while the nodes in (x∩y∩z) receive (*m* + 3) values including *m* raw keys and three nonces $\left(\overset{m\;\mathrm{keys}}{\overset{︷}{K_{i},\;1\leq i\leq m}},\overset{3\;\mathrm{nonce}}{\overset{︷}{r_{x},r_{y},r_{z}}}\right)$. Finally, as shown in Figure 5, nodes #5, #6, and #7 in areas *A*_5_, *A*_6_, and *A*_7_ have a key ring of size *m*; nodes #2, #3, and #4 in areas *A*_2_, *A*_3_, and *A*_4_ have a ring size of (*m* + 2); and node #1 in area *A*_1_ has a key ring size of (*m* + 3). However, the key ring sizes of I^2^KPDP are 3*m* for *A*_1_ and 2*m* for *A*_2_, *A*_3_, and *A*_4_.

Suppose a key $K_{ID}$ with an identifier *ID* is selected from *S* given to a node. If node #a exists in the group (x−(x∩y)−(x∩z)), the node will receive the hashed key $f(K_{ID}+r_{x})$. Suppose another node is also in (x−(x∩y)−(x∩z)). It may share the common key $f(K_{ID}+r_{x})$ with node #a if it also has the same hashed key from key pool *S*. Consider the case that node #b is in (x∩y), (x∩z), or (x∩y∩z) and has a key with the same identifier. It receives the raw key $K_{ID}$ with the nonce $\left(r_{x},\;r_{y}\right)$, $\left(r_{x},\;r_{z}\right)$, or $\left(r_{x},\;r_{y},\;r_{z}\right)$. For these cases, node #b has the nonce $r_{x}$, and it can calculate the hashed key $f(K_{ID}+r_{x})$ to share the common key with node #a. In I^2^KPSP, we use an extra nonce to share the pairwise key between nodes.

In the same group *x*, both nodes share exactly *l* independent keys from the key pool *S* that have the probability $P_{Share}^{\left(x\right)}(l)$ like in I^2^KPDP. Suppose two nodes in the intersected group (x∩y) have one same key $K_{ID}$; they could have the hashed keys $f(K_{ID}+r_{x})$ and $f(K_{ID}+r_{y})$ for establishing links. Thus, the probabilities that two nodes have exactly *l* independent keys in intersected groups are $\left(\begin{array}{c} \left|S\right|\\ l/2 \end{array}\right)\left(\begin{array}{c} |S|-l/2\\ 2(m-l/2) \end{array}\right)\left(\begin{array}{c} 2(m-l/2)\\ m-l/2 \end{array}\right)/{\left(\begin{array}{c} \left|S\right|\\ m \end{array}\right)}^{2}$, on which the $P_{Share}^{\left(x\cap y\right)}(l)$ and $P_{Share}^{\left(x\cap y\cap z\right)}(l)$ of I^2^KPSP are derived in Equation (13).

(13) $$\left\{\begin{array}{l} P_{Share}^{\left(x\cap y\right)}(l)=P_{Share}^{\left(x\right)}(l/2),\;\mathrm{where}\;l\;\mathrm{is}\;\mathrm{even}\\ P_{Share}^{\left(x\cap y\cap z\right)}(l)=P_{Share}^{\left(x\right)}(l/3),\;\mathrm{where}\;l\;\mathrm{is}\;a\;\mathrm{multiple}\;\mathrm{of}\;3 \end{array}\right.$$

Same as I^2^KPDP, we use $P_{Share}^{\left(1\right)}(l)$, $P_{Share}^{\left(2\right)}(l)$, and $P_{Share}^{\left(3\right)}(l)$ to represent the probabilities for single-intersected, double-intersected, and triple-intersected groups. Then, for our I^2^KPSP, we can get $P_{Share}^{\left(A\right)}(l)$, $P_{Share}^{\left(B\right)}(l)$, and $P_{Share}^{\left(C\right)}(l)$ using the keys from the same key pool *S* with different nonces $x_{A}$, $x_{B}$, and $x_{C}$. Consider the same case $n_{1}=n_{2}=n_{3}=n_{4}=n/8$ in I^2^KPDP. The $P_{Share}^{\left(A\right)}(l)$ for I^2^KPSP is derived in Equation (14), which is approximated to $P_{Share}^{\left(A\right)}(l)\approx P_{Share}^{\left(1\right)}(l).$

(14) $$\left\{\begin{array}{l} P_{Share}^{\left(A\right)}(l)=\left(\begin{array}{c} \left(n_{5}\times (2n-n_{5})+2n_{2}n_{3}\right)\cdot P_{Share}^{\left(1\right)}(l)+\left(n_{2}^{2}+n_{3}^{2}\right.\\ \left.+2n_{1}(n_{2}+n_{3})\right)\cdot P_{Share}^{\left(1\right)}(l/2)+n_{1}^{2}\cdot P_{Share}^{\left(1\right)}(l/3) \end{array}\right)/n^{2}\\ =\left(57P_{Share}^{\left(1\right)}(l)+6P_{Share}^{\left(1\right)}(l/2)+P_{Share}^{\left(1\right)}(l/3)\right)/64 \end{array}\right.$$

We can also prove that I^2^KPSP satisfies intragroup and intergroup conditions, and we calculate the probabilities of a link being compromised: $P_{Link-Com}^{\left(A\to G\right)}$, $P_{Link-Com}^{\left(B\to G\right)}$, and $P_{Link-Com}^{\left(C\to G\right)}$ (same as Theorem 2). Although I^2^KPSP does not use a hash chain, it uses a hash function *f*(·) to generate hashed keys in the area x−(x∩y)−(x∩z). We use the approximation $P_{Share}^{\left(x\right)}(l)\approx P_{Share}^{\left(1\right)}(l)$ in Lemma 1, i.e., we do not consider the intersected groups $P_{Share}^{\left(2\right)}(l)$. For $n_{1}=n_{2}=n_{3}=n_{4}=n/8$, we have $P_{Share}^{\left(x\right)}(l)=\left(57P_{Share}^{\left(1\right)}+6P_{Share}^{\left(2\right)}+P_{Share}^{\left(3\right)}\right)/64$. This implies the fractions of intersected groups $P_{Share}^{\left(2\right)}(l)$ and $P_{Share}^{\left(3\right)}(l)$ are very small. In fact, there is already a large portion $57/64\cdot P_{Share}^{\left(1\right)}(l)$ in Equation (14) for $n_{1}=n_{2}=n_{3}=n_{4}=n/8$, and thus we consider using $P_{Share}^{\left(1\right)}(l)$ only. Four cases using $P_{Share}^{\left(1\right)}(l)$ in group *A* as deriving $P_{Share}^{\left(A\right)}(l)$ are (i) node *i* in $A_{1}$ and node *j* in $A_{5}$, (ii) node *i* in $A_{2}$ and node *j* in $A-(A_{1}\cup A_{2})$, (iii) node *i* in $A_{3}$ and node *j* in $A-(A_{1}\cup A_{3})$, and (iv) node *i* in $A_{5}$ and node *j* in *A*. Two hash operations are required when nodes *i* and *j* are in $A_{2}$ and $A_{3}$, respectively. Note: even though two nodes have raw keys, we use a hashed key to establish a link for keeping consistency in the whole group. The above concludes that the average number of hash operations per node to share a common key in group *A* is $N_{ave}^{\left(A\right)}=$ $\left(2n_{5}(n-n_{5})+4n_{2}n_{3}\right)/n^{2}$ (see Equation (15)).

(15) $$\left\{\begin{array}{l} N_{avg}^{\left(A\right)}=\frac{1}{n^{2}}\cdot (\overset{\mathrm{node}\;i\;\mathrm{in}\;A_{1}\;\mathrm{node}\;j\;\mathrm{in}\;A_{5}}{\overset{︷}{1\;\mathrm{hash}\;\mathrm{ops}}}+\overset{\mathrm{node}\;i\;\mathrm{in}\;A_{2}\;\mathrm{node}\;j\;\mathrm{in}\;A-(A_{1}\cup A_{2})}{\overset{︷}{\overset{i\;\mathrm{in}\;A_{2}\;j\;\mathrm{in}\;A_{5}}{\overset{︷}{1\;\mathrm{hash}\;\mathrm{ops}}}+\overset{i\;\mathrm{in}\;A_{2}\;j\;\mathrm{in}\;A_{3}}{\overset{︷}{2\;\mathrm{hash}\;\mathrm{ops}}}}}+\overset{\mathrm{node}\;i\;\mathrm{in}\;A_{3}\;\mathrm{node}\;j\;\mathrm{in}\;A-(A_{1}\cup A_{3})}{\overset{︷}{\overset{i\;\mathrm{in}\;A_{3}\;j\;\mathrm{in}\;A_{5}}{\overset{︷}{1\;\mathrm{hash}\;\mathrm{ops}}}+\overset{i\;\mathrm{in}\;A_{3}\;j\;\mathrm{in}\;A_{2}}{\overset{︷}{2\;\mathrm{hash}\;\mathrm{ops}}}}}\\ +\overset{\mathrm{node}\;i\;\mathrm{in}\;A_{5}\;\mathrm{node}\;j\;\mathrm{in}\;A}{\overset{︷}{\overset{i\;\mathrm{in}\;A_{5}\;j\;\mathrm{in}\;A_{5}}{\overset{︷}{0\;\mathrm{hash}\;\mathrm{ops}}}+\overset{i\;\mathrm{in}\;A_{5}\;j\;\mathrm{in}\;(A-A_{5})}{\overset{︷}{1\;\mathrm{hash}\;\mathrm{ops}}}}})=\left(2n_{5}(n-n_{5})+4n_{2}n_{3}\right)/n^{2} \end{array}\right.$$

By the same argument, the average numbers of hash operations are $N_{avg}^{\left(B\right)}=$ $\left(2n_{6}(n-n_{6})+4n_{2}n_{4}\right)/n^{2}$ and $N_{avg}^{\left(C\right)}=\left(2n_{6}(n-n_{6})+4n_{2}n_{4}\right)/n^{2}$ for groups *B* and *C*. Each node in group *x* performs the hash function *f*(·) an average of $N_{avg}^{\left(x\right)}$ times on all the *l* shared keys where *l* ≥ *q*. Since $P_{Share}^{\left(1\right)}(l)/P_{Link-Est}^{\left(1\right)}$ is the probability that an established link is secured with *l* keys for a single-intersected group, the total average number $N_{I^{2}\mathrm{KPSP}}^{\left(x\right)},\;x\in \{A,\;B\;,C\}$ of hash operations in group *x* for *q*-composite I^2^KPSP is calculated in Equation (16).

(16) $$\left\{\begin{array}{l} N_{I^{2}\mathrm{KPSP}}^{\left(x\right)}=N_{avg}^{\left(x\right)}\times \sum_{l=q}^{m}\left(l\times \frac{P_{Share}^{\left(1\right)}(l)}{\sum_{l=q}^{m}P_{Share}^{\left(1\right)}(l)}\right),\;\mathrm{where}\;x\in \{A,\;B,\;C\},\;N_{avg}^{\left(A\right)}=\left(2n_{5}(n-n_{5})+4n_{2}n_{3}\right)/n^{2}\\ ,\;N_{avg}^{\left(B\right)}=\left(2n_{6}(n-n_{6})+4n_{2}n_{4}\right)/n^{2}\;\mathrm{and}\;N_{avg}^{\left(C\right)}=\left(2n_{7}(n-n_{7})+4n_{3}n_{4}\right)/n^{2} \end{array}\right.$$

I^2^KPDP and I^2^KPSP do not have resiliency against LNAs, since attackers may know the nodes belong to the intersected groups from their locations. By an LNA, attackers could intentionally capture *k* nodes in the intersected groups *A*_1_, *A*_2_, *A*_3_, and *A*_4_. Theorem 3 shows all the probabilities $P_{Link-Com}^{\left(A_{1}\to G\right)}$, $P_{Link-Com}^{\left(A_{2}\to G\right)}$, and $P_{Link-Com}^{\left(A_{3}\to G\right)}$ greater than $P_{Link-Com}^{\left(A\to G\right)}$, and this means that an LNA is more severe and damaging than an NA. When compared with the case where *k* nodes are captured in a random fashion in group *A*, the captured nodes in the intersected groups *A*_1_, *A*_2_, and *A*_3_ aggravate the NA attack.

**Theorem** **3.** *In q-composite I^2^KPDP and I^2^KPSP, when k nodes are captured randomly in the intersected groups $A_{1}$, $A_{2}$, and $A_{3}$ (i.e., using LNA), the probabilities $P_{Link-Com}^{\left(A_{1}\to G\right)}$, $P_{Link-Com}^{\left(A_{2}\to G\right)}$* *, and $P_{Link-Com}^{\left(A_{3}\to G\right)}$ of a link being compromised are greater than the probability $P_{Link-Com}^{\left(A\to G\right)}$.*

**Proof.**  Suppose *k* captured nodes are in $A_{1}$, and there are $C_{k}^{n_{1}}$ types to select *k* captured nodes. Because of using an LNA, attackers could capture all these *k* nodes in $A_{1}$. Thus, based on the notion of Equation (12), we can derive $P_{Link-Com}^{\left(A_{1}\to G\right)}$, $P_{Link-Com}^{\left(A_{2}\to G\right)}$, and $P_{Link-Com}^{\left(A_{3}\to G\right)}$ as follows.

(17) $$\left\{\begin{array}{l} P_{Link-Com}^{\left(A_{1}\to G\right)}\approx \frac{1}{3}\cdot (\overset{\begin{array}{c} \mathrm{attacker}\;\mathrm{intentionally}\\ \mathrm{capture}\;k\;\mathrm{nodes}\;\mathrm{in}\;A_{1} \end{array}}{\overset{︷}{\frac{C_{k}^{n_{1}}}{C_{k}^{n_{1}}}}}\cdot \overset{\mathrm{compromise}\;\mathrm{the}\;\mathrm{links}\;\mathrm{in}\;\mathrm{three}\;\mathrm{groups}\;A,\;B\;\mathrm{and}\;C}{\overset{︷}{3\left(\sum_{l=q}^{m}{\left(1-{\left(1-\frac{m}{\left|S\right|}\right)}^{k}\right)}^{l}\cdot \frac{P_{Share}^{\left(1\right)}(l)}{\sum_{l=q}^{m}P_{Share}^{\left(1\right)}(l)}\right)}})=\left(\sum_{l=q}^{m}{\left(1-{\left(1-\frac{m}{\left|S\right|}\right)}^{k}\right)}^{l}\cdot \frac{P_{Share}^{\left(1\right)}(l)}{\sum_{l=q}^{m}P_{Share}^{\left(1\right)}(l)}\right)\\ P_{Link-Com}^{\left(A_{2}\to G\right)}\approx \frac{1}{3}\cdot (\overset{\begin{array}{c} \mathrm{attacker}\;\mathrm{intentionally}\\ \mathrm{capture}\;k\;\mathrm{nodes}\;\mathrm{in}\;A_{2} \end{array}}{\overset{︷}{\frac{C_{k}^{n_{2}}}{C_{k}^{n_{2}}}}}\cdot \overset{\mathrm{compromise}\;\mathrm{the}\;\mathrm{links}\;\mathrm{in}\;\mathrm{two}\;\mathrm{groups}\;A\;\mathrm{and}\;B}{\overset{︷}{2\left(\sum_{l=q}^{m}{\left(1-{\left(1-\frac{m}{\left|S\right|}\right)}^{k}\right)}^{l}\cdot \frac{P_{Share}^{\left(1\right)}(l)}{\sum_{l=q}^{m}P_{Share}^{\left(1\right)}(l)}\right)}})=\frac{2}{3}\left(\sum_{l=q}^{m}{\left(1-{\left(1-\frac{m}{\left|S\right|}\right)}^{k}\right)}^{l}\cdot \frac{P_{Share}^{\left(1\right)}(l)}{\sum_{l=q}^{m}P_{Share}^{\left(1\right)}(l)}\right)\\ P_{Link-Com}^{\left(A_{3}\to G\right)}\approx \frac{1}{3}\cdot (\overset{\begin{array}{c} \mathrm{attacker}\;\mathrm{intentionally}\\ \mathrm{capture}\;k\;\mathrm{nodes}\;\mathrm{in}\;A_{3} \end{array}}{\overset{︷}{\frac{C_{k}^{n_{3}}}{C_{k}^{n_{3}}}}}\cdot \overset{\mathrm{compromise}\;\mathrm{the}\;\mathrm{links}\;\mathrm{in}\;\mathrm{two}\;\mathrm{groups}\;A\;\mathrm{and}\;C}{\overset{︷}{2\left(\sum_{l=q}^{m}{\left(1-{\left(1-\frac{m}{\left|S\right|}\right)}^{k}\right)}^{l}\cdot \frac{P_{Share}^{\left(1\right)}(l)}{\sum_{l=q}^{m}P_{Share}^{\left(1\right)}(l)}\right)}})=\frac{2}{3}\left(\sum_{l=q}^{m}{\left(1-{\left(1-\frac{m}{\left|S\right|}\right)}^{k}\right)}^{l}\cdot \frac{P_{Share}^{\left(1\right)}(l)}{\sum_{l=q}^{m}P_{Share}^{\left(1\right)}(l)}\right) \end{array}\right.$$

Via Equation (12), we have $P_{Link-Com}^{\left(A\to G\right)}\approx \left(\frac{C_{k}^{n}+C_{k}^{n_{1}+n_{2}}+C_{k}^{n_{1}+n_{3}}}{3C_{k}^{n}}\right)\cdot \left(\sum_{l=q}^{m}{\left(1-{\left(1-\frac{m}{\left|S\right|}\right)}^{k}\right)}^{l}\cdot \frac{P_{Share}^{\left(1\right)}(l)}{\sum_{l=q}^{m}P_{Share}^{\left(1\right)}(l)}\right)$. Since $n_{1}<<n$, $n_{2}<<n$, and $n_{3}<<n$, $(C_{k}^{n_{1}+n_{2}}+C_{k}^{n_{1}+n_{3}})<C_{k}^{n}$. Finally, we have $P_{Link-Com}^{\left(A_{1}\to G\right)}$, $P_{Link-Com}^{\left(A_{2}\to G\right)}$, and $P_{Link-Com}^{\left(A_{3}\to G\right)}$ greater than $P_{Link-Com}^{\left(A\to G\right)}$. □

The above demonstrates that I^2^KPDP and I^2^KPSP do not have resiliency against LNAs. In addition, for I^2^KPSP, the nodes in intersected groups store the nonce in plaintext type. The attacker can capture only one node to obtain the nonce. Thus, if an attacker compromises one node in area *A*_1_ to get *r_C_*, he can capture nodes in *A*_2_ for raw keys and then compromise the link in group *C* with the compromised raw keys in *A*_2_. To effectively tackle LNAs and solve the above problem in I^2^KPSP, we adopt a hash chain to design I^2^KPHC.

### 4.3. The Proposed I^2^KPHC

**Figure 6:**
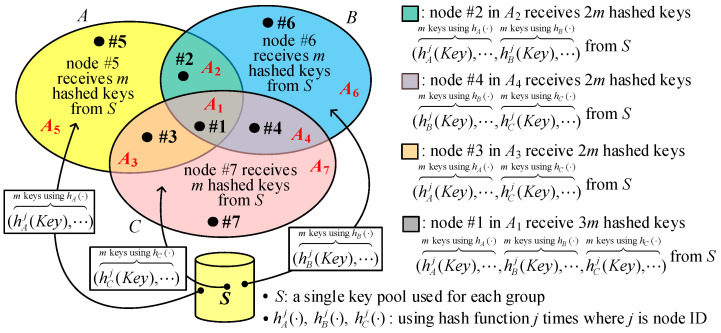
The proposed I^2^KPHC.

We adopt the hash chain approach in [7] and, in the meantime, give the nodes in intersected groups the large node identifier “*j*” to resist LNAs, where 1 ≤ *j* ≤ *n* and the value *n* is the group size (the number of nodes for each group). In fact, we can use different key pools like in I^2^KPDP or a single pool like in I^2^KPSP to design the proposed I^2^KPHC. When using different key pools, we just need one hash function, while we need different hash functions for different groups when using one key pool. We also use three groups, *A*, *B*, and *C*, to describe I^2^KPHC. As illustrated in Figure 6, we adopt one key pool *S* and three hash functions, $h_{A}(\cdot )$, $h_{B}(\cdot )$, and $h_{C}(\cdot )$, for each group to describe our I^2^KPHC.

Consider a node with ID “*j*” for the following cases: (i) in group (x−(x∩y)−(x∩z)), (ii) in group (x∩y), and (iii) in group (x∩y∩z), where *x*, *y*, *z*∈{*A*, *B*, *C*}. Suppose *m* random keys (say $K_{1}\sim K_{m}$) are selected from key pool *S*, then *m* hashed keys $\left(\overset{m\;\mathrm{hashed}\;\mathrm{keys}\;\mathrm{using}\;h_{x}(\cdot )}{\overset{︷}{h_{x}^{j}(K_{1})\sim h_{x}^{j}(K_{m})}}\right)$ are given to node #*j* in group (x−(x∩y)−(x∩z)). There are 2*m* hashed keys $\left(\overset{m\;\mathrm{hashed}\;\mathrm{keys}\;\mathrm{using}\;h_{x}(\cdot )}{\overset{︷}{h_{x}^{j}(K_{1})\sim h_{x}^{j}(K_{m})}},\overset{m\;\mathrm{hashed}\;\mathrm{keys}\;\mathrm{using}\;h_{y}(\cdot )}{\overset{︷}{h_{y}^{j}(K_{1})\sim h_{y}^{j}(K_{m})}}\right)$ and 3*m* hashed keys $(\overset{m\;\mathrm{hashed}\;\mathrm{keys}\;\mathrm{using}\;h_{x}(\cdot )}{\overset{︷}{h_{x}^{j}(K_{1})\sim h_{x}^{j}(K_{m})}},$ $\overset{m\;\mathrm{hashed}\;\mathrm{keys}\;\mathrm{using}\;h_{y}(\cdot )}{\overset{︷}{h_{y}^{j}(K_{1})\sim h_{y}^{j}(K_{m})}},\overset{m\;\mathrm{hashed}\;\mathrm{keys}\;\mathrm{using}\;h_{z}(\cdot )}{\overset{︷}{h_{z}^{j}(K_{1})\sim h_{z}^{j}(K_{m})}})$ given to node #*j* in the intersected groups (x∩y) and (x∩y∩z), respectively. Finally, as shown in Figure 5, nodes #5, #6, and #7 in areas *A*_5_, *A*_6_ and *A*_7_ have a ring size of *m*; nodes #2, #3, and #4 in areas *A*_2_, *A*_3_, and *A*_4_ have a ring size of 2*m*; and node #7 in *A*_1_ area has a ring size of 3*m*.

Suppose a key $K_{ID}$ with an identifier *ID* is selected from *S* given to a node with node ID “$j_{1}$”. If the node is in the group (x−(x∩y)−(x∩z)), it receives the key $h_{x}^{j_{1}}(K_{ID})$. Suppose another node with node ID “$j_{2}$” is also in the group (x−(x∩y)−(x∩z)); it has $h_{x}^{j_{2}}(K_{ID})$. When $j_{1}>j_{2}$, the node #$j_{2}$ applies the hash function $h_{x}(\cdot )$ $\left(j_{1}-j_{2}\right)$ times on $h_{x}^{j_{2}}(K_{ID})$, and then both nodes may share a common key $h_{x}^{j_{1}}(K_{ID})$. Consider other cases for node #$j_{2}$ in (i) (x∩y), (ii) (x∩z), (iii) (x∩y∩z), (iv) (y−(x∩y)−(y∩z)), and (v) (z−(y∩z)−(x∩z)). For cases (i), (ii), and (iii), node #$j_{2}$ also has $h_{x}^{j_{2}}(K_{ID})$ such that it can share the same key $h_{x}^{j_{1}}(K_{ID})$ with node #$j_{1}$. However, node #$j_{2}$ for cases (iv) and (v) only has $h_{y}^{j_{2}}(K_{ID})$ and $h_{z}^{j_{2}}(K_{ID})$, respectively, and thus it cannot share the common key with the node #$j_{1}$. Via the above observations, the approach using the hash function does not affect establishing a link, but it needs extra hash operations when establishing secure links. Although the I^2^KPHC in Figure 6 uses one key pool for all groups, it adopts different hash functions for different groups. All the properties of I^2^KPHC are more similar to those of I^2^KPSP, because both approaches use one key pool. However, I^2^KPHC has the same key ring sizes as I^2^KPDP. The probabilities $P_{Share}^{\left(x\right)}(l)$, $P_{Share}^{\left(x\cap y\right)}(l)$, $P_{Share}^{\left(x\cap y\cap z\right)}(l)$, $P_{Link-Est}^{\left(x\right)}$, $P_{Link-Est}^{\left(x\cap y\right)}$, and $P_{Link-Est}^{\left(x\cap y\cap z\right)}$, where *x*, *y*, and *z*∈{*A*, *B*, *C*}, are the same as in I^2^KPSP. And, the proposed I^2^KPHC also satisfies intragroup and intergroup conditions.

**Figure 7:**
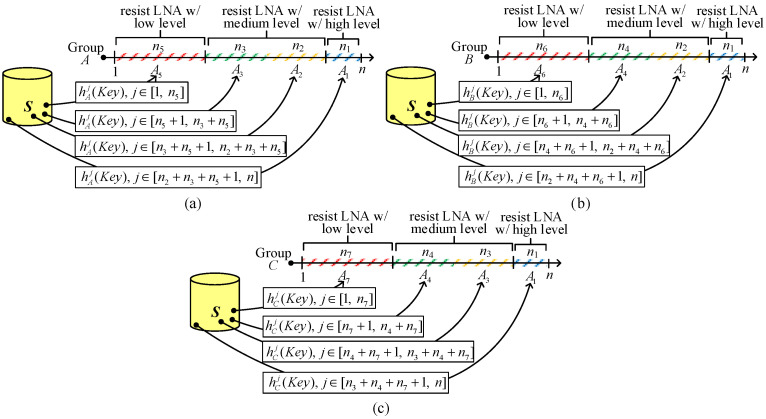
Key selections in I^2^KPHC to resist LNA for (**a**) group *A*, (**b**) group *B*, and (**c**) group *C*.

When compared with I^2^KPDP and I^2^KPDSP, the major difference of I^2^KPHC is resiliency against LNAs. LNA resiliency based on the hash chain is described as follows. The diagrammatic representation of key selection rules is illustrated in Figure 7. The number of nodes for each group is *n* (|A|=|B|=|C|=*n*). For simplicity, we use 1~*n* to represent the node *ID* “*j*”, where 1 ≤ *j* ≤ *n*. How to choose an encoded *ID* for sensor nodes for disjointed areas in groups *A*, *B,* and *C* to resist LNAs is given in Figure 7a–c, respectively.

Consider the key selections for group *A*, which is composed of four geometrically disjointed areas (*A*_1_, *A*_2_, *A*_3_, *A*_5_). From I^2^KPDP and I^2^KPSP, it is observed that the captured nodes in intersected groups, e.g., in (x∩y∩z) and (x∩y), the NA will be seriously aggravated. Therefore, we define three levels of resiliency (high, medium, low) against NAs for the intersected groups: *A*_1_ (triple-intersected), *A*_2_ and *A*_3_ (double-intersected), and *A*_5_ (single-intersected)). We use medium resiliency for *A*_2_ and *A*_3_, because both areas have probabilities of compromised links in (A∩B) and (A∩C), respectively. The reason choosing high resiliency for *A*_1_ is to resist the high probability of compromised links. The key selection for group *A* is illustrated in Figure 7a. We use the *ID* $j\in [n_{2}+n_{3}+n_{5}+1,\;n]$ to prepare a hashed key $h_{A}^{j}(Key)$ for $A_{1}$, the *ID* $j\in [n_{2}+n_{5}+1,$ $n_{2}+n_{3}+n_{5}]$ to prepare a hashed key $h_{A}^{j}(Key)$ for *A*_2_, the *ID* $j\in [n_{5}+1,\;n_{2}+n_{5}]$ to prepare a hashed key $h_{A}^{j}(Key)$ for *A*_3_, and the *ID* $j\in [1,\;n_{5}]$ to prepare a hashed key $h_{A}^{j}(Key)$ for *A*_5_. When the nodes in *A*_1_ are captured, an attacker has the hashed key $h_{A}^{j_{1}}(Key)$, where $j_{1}\in [n_{2}+n_{3}+n_{5}+1,\;n]$, but he cannot obtain the hashed key $h_{A}^{j_{2}}(Key)$ for node $\#j_{2}$ in areas *A*_2_, *A*_3_, and *A*_5_ because $j_{1}>(n_{2}+n_{3}+n_{5})\geq j_{2}$. We can also check another situation where if node $\#j_{1}$ is in areas *A*_2_ and *A*_3_ and node $\#j_{2}$ is in area $A_{5}$, both nodes $\#j_{1}$ and $\#j_{2}$ cannot share the common key. This is because $j_{1}\in [n_{5}+1,\;n_{2}+n_{3}+n_{5}]$ is greater than $j_{2}\in [1,\;n_{5}]$. Obviously, node $\#j_{1}$ in *A*_5_ only has the probability to obtain the same hashed key $h_{A}^{j_{2}}(Key)$ with node $\#j_{2}$ also located in *A*_5_. By the same argument, we can choose the hashed keys for sensor nodes in groups *B* and *C*. The key selections for groups *B* (including *A*_1_, *A*_2_, *A*_4_, and *A*_6_) and *C* (including *A*_1_, *A*_3_, *A*_4_, and *A*_7_) are shown in Figure 7b,c, respectively.

In [7], a parameter key-chain length *L* is introduced to reduce these hash operations. Each node #*j* applies one-way hash operation to their keys *j* (mod *L*) times instead of *j* times, namely storing ${h_{x}^{j}}^{\;(\mathrm{mod}\;L)}(Key)$ instead of $h_{x}^{j}(Key)$. Finally, the number of hash operations for establishing a pairwise key is bounded by *L*. When adopting the notion of key-chain length *L* in I^2^KPHC to save energy consumption overhead, the key selections in Figure 7a–c should be a little modified. Here, we only use key selection for group *A* to describe the modification. We first define $L_{1},\;L_{2},\;L_{3},\;\mathrm{and}\;L_{5}$, where $L=L_{1}+L_{2}+L_{3}+L_{5}$. The choice of these values can be determined according to the group size *n* and the reduction in computation overhead. After the key selection for group *A* in Figure 7a, let the node *ID* $j_{A_{1}}\in [n_{2}+n_{3}+n_{5}+1,\;n]$, $j_{A_{2}}\in [n_{2}+n_{5}+1,\;n_{2}+n_{3}+n_{5}]$, $j_{A_{3}}\in$ $\left[n_{2}+n_{5}+1,\;n_{2}+n_{3}+n_{5}\right]$, and $j_{A_{5}}\in [n_{5}+1,\;n_{2}+n_{5}]$. Next, we figure out the new modified ID ${j^{\prime }}_{A_{1}}$, ${j^{\prime }}_{A_{2}}$, ${j^{\prime }}_{A_{3}}$, and ${j^{\prime }}_{A_{5}}$ by Equation (18a). By the same argument, we may choose $L_{4},\;L_{6}\;\mathrm{and}\;L_{7}$ for $A_{4},\;A_{6}\;\mathrm{and}\;A_{7}$, and the new modified ID for groups *B* and *C* are given in Equations (18b) and (18c), respectively.


(18a) $$\left\{\begin{array}{l} {j^{\prime }}_{A_{1}}=j_{A_{1}}\;(\mathrm{mod}\;L_{1})+(L_{2}+L_{3}+L_{5});{j^{\prime }}_{A_{2}}=j_{A_{2}}\;(\mathrm{mod}\;L_{2})+(L_{3}+L_{5})\\ {j^{\prime }}_{A_{3}}=j_{A_{3}}\;(\mathrm{mod}\;L_{3})+L_{5};{j^{\prime }}_{A_{5}}=j_{A_{5}}\;(\mathrm{mod}\;L_{5}) \end{array}\right.$$

(18b) $$\left\{\begin{array}{l} {j^{\prime }}_{A_{1}}=j_{A_{1}}\;(\mathrm{mod}\;L_{1})+(L_{2}+L_{4}+L_{6});{j^{\prime }}_{A_{2}}=j_{A_{2}}\;(\mathrm{mod}\;L_{2})+(L_{4}+L_{6})\\ {j^{\prime }}_{A_{4}}=j_{A_{4}}\;(\mathrm{mod}\;L_{4})+L_{6};{j^{\prime }}_{A_{6}}=j_{A_{6}}\;(\mathrm{mod}\;L_{6}) \end{array}\right.$$

(18c) $$\left\{\begin{array}{l} {j^{\prime }}_{A_{1}}=j_{A_{1}}\;(\mathrm{mod}\;L_{1})+(L_{3}+L_{4}+L_{7});{j^{\prime }}_{A_{3}}=j_{A_{3}}\;(\mathrm{mod}\;L_{3})+(L_{4}+L_{7})\\ {j^{\prime }}_{A_{4}}=j_{A_{4}}\;(\mathrm{mod}\;L_{4})+L_{7};{j^{\prime }}_{A_{7}}=j_{A_{7}}\;(\mathrm{mod}\;L_{7}) \end{array}\right.$$

We herein use the original node ID to more easily analyze the LNA resiliency of our I^2^KPHC. Actually, both have similar results. Lemma 2 shows the probability that a given key is known in the area $A_{j}$ when a node is randomly captured in the area $A_{i}$. And, the probability $\alpha_{A_{i}\to A_{j}}$, where $0\leq \alpha_{A_{i}\to A_{j}}\leq 1$, is referred to as an improvement in I^2^KPHC. Since each node has *m* keys from key pool *S*, the probability that a key has been discovered in $A_{j}$ when a node is compromised in $A_{i}$ is $\left(\alpha_{A_{i}\to A_{j}}\cdot m/\left|S\right|\right)$ for I^2^KPHC. Thus, the value $\alpha_{A_{i}\to A_{j}}=1$ implies no improvement from using a hash chain, i.e., the probabilities of compromised links are unchanged. On the other hand, $\left(\alpha_{A_{i}\to A_{j}}\cdot m/\left|S\right|\right)$ is 0 for $\alpha_{A_{i}\to A_{j}}=0$. Namely, the approach can completely tackle LNAs. The value $\alpha_{A_{i}\to A_{j}}$ ranging from 1 to 0 shows the attack mitigation of LNAs. A hash chain could reduce interception from the attacker, and that is why we call the value $\alpha_{A_{i}\to A_{j}}$ “improvement”.

**Lemma** **2.** *When using the hash chain class with n* ($h_{x}^{j}(Key),\;1\leq j\leq n$ *) in I^2^KPHC, the probability $\alpha_{A_{i}\to A_{j}}$* *is derived as follows.*

(19) $$\left\{\begin{array}{l} \alpha_{A_{i}\to A_{j}}=\left(n_{i}+1\right)/\left(2n_{i}\right);\;\mathrm{if}\;A_{i}=A_{j}\\ \alpha_{A_{i}\to A_{j}}=0\;;\;\mathrm{if}\;(\mathrm{node}\;\mathrm{ID}\;j_{1}\in A_{i}>j_{2}\in A_{j})\vee (\mathrm{no}\;\mathrm{same}\;\mathrm{hash}\;\mathrm{function}\;\mathrm{in}\;A_{i}\;\mathrm{and}\;A_{j})\\ \alpha_{A_{i}\to A_{j}}=1\;;\;\mathrm{if}\;(\mathrm{node}\;\mathrm{ID}\;j_{1}\in A_{i}\leq j_{2}\in A_{j})\wedge (\mathrm{one}\;\mathrm{same}\;\mathrm{hash}\;\mathrm{function}\;\mathrm{in}\;A_{i}\;\mathrm{and}\;A_{j}) \end{array}\right.$$

**Proof.**  For the case $A_{i}=A_{j}$ with $|A_{i}|=n_{i}$ nodes in this area, suppose the node *ID* in $A_{i}$ ranges between $n_{x}$ and $\left(n_{x}+n_{i}-1\right)$. For any hashed key stored in the nodes in $A_{i}$, it is initially hashed $\left(n_{x}+w\right)$ times, where $0\leq w\leq (n_{i}-1)$, with a probability $1/n_{i}$. When a captured node is hashed $\left(n_{x}+w\right)$ times, the probability that a key has the same identifier can be figured out is $(n_{i}-w)/n_{i}$ (note: a hash function can only be accomplished forwardly). Thus, the probability to find a key having the same identifier with a compromised node is $\sum_{w=0}^{n_{i}-1}1/n_{i}\cdot (n_{i}-w)/n_{i}=(n_{i}+1)/\left(2n_{i}\right).$ Consider the case “(node ID *j*_1_∈*A_i_* > *j*_2_∈*A_j_*)∨ (no same hash function in *A_i_* and *A_j_*)”. Firstly, the condition $j_{1}>j_{2}$ implies that even though both areas have same hash function $h_{x}(\cdot )$, the hashed key $h_{x}^{j_{1}}(Key)$ cannot be disclosed from $h_{x}^{j_{2}}(Key)$. Secondly, both areas with different hash function cannot have the same hashed key. Thus, we have $\alpha_{A_{i}\to A_{j}}=0$ for this case. Consider the other case “(node ID *j*_1_∈*A_i_* ≤ *j*_2_∈*A_j_*) ∧ (one same hash function in *A_i_* and *A_j_*)”. Both areas have the same hash function $h_{x}(\cdot )$. Since $j_{1}$ ≤ $j_{2}$, the hashed key $h_{x}^{j_{1}}(Key)$ can be derived from $h_{x}^{j_{2}}(Key)$. This implies $\alpha_{A_{i}\to A_{j}}=1$. □

By LNAs, attackers could intentionally capture the nodes in intersected groups (*A*_1_~*A*_4_) to compromise the links. Consider intersected groups *A*_1_, *A*_2_, and *A*_3_ in group *A* (note: the analysis for *A*_4_ is the same as for *A*_2_ and *A*_3_ and is omitted here). In Theorem 4, the probabilities $P_{Link-Com}^{\left(A_{1}\to G\right)}$, $P_{Link-Com}^{\left(A_{2}\to G\right)}$, and $P_{Link-Com}^{\left(A_{3}\to G\right)}$ of I^2^KPHC are derived, where $P_{Link-Com}^{\left(A_{1}\to G\right)}$ and $P_{Link-Com}^{\left(A_{2}\to G\right)}$ are lesser than those in I^2^KPDP and I^2^KPSP, and $P_{Link-Com}^{\left(A_{2}\to G\right)}$ is almost the same. The result implies that the resiliency of I^2^KPHC against LNAs is enhanced.

**Theorem** **4.** *In q-composite I^2^KPHC, k nodes are captured randomly in the intersected groups A_1_, A_2_, and A_3_ (i.e., using LNA). When compared with I^2^KPDP and I^2^KPSP, I^2^KPHC has smaller* $P_{Link-Com}^{\left(A_{1}\to G\right)}$*and $P_{Link-Com}^{\left(A_{2}\to G\right)}$and almost the same $P_{Link-Com}^{\left(A_{3}\to G\right)}$*.

**Proof.**  Same as deriving the probabilities in Theorem 3, because there is already a large portion $57/64\cdot P_{Share}^{\left(1\right)}(l)$, we consider using $P_{Share}^{\left(1\right)}(l)$ only. Four cases using $P_{Share}^{\left(1\right)}(l)$ in group *A* as deriving $P_{Share}^{\left(A\right)}(l)$ are (i) node *i* in *A*_1_ and node *j* in *A*_5_, (ii) node *i* in *A*_2_ and node *j* in $A-(A_{1}\cup A_{2})$, (iii) node *i* in *A*_3_ and node *j* in $A-(A_{1}\cup A_{3})$, and (iv) node *i* in *A*_5_ and node *j* in *A*. In case (i), two nodes can share a hashed key $h^{i}(Key)$ since *i* > *j*. Thus, the link may be compromised with the probability $\sum_{l=q}^{m}{\left(1-{\left(1-\alpha_{A_{1}\to A_{1}}\frac{m}{\left|S\right|}\right)}^{k}\right)}^{l}\cdot \frac{P_{Share}^{\left(1\right)}(l)}{\sum_{l=q}^{m}P_{Share}^{\left(1\right)}(l)}$ with the improvement $\alpha_{A_{1}\to A_{1}}=\left(n_{1}+1\right)/2n_{1}$. For cases (ii) and (iii), the improvements $\alpha_{A_{1}\to A_{2}}=\alpha_{A_{1}\to A_{3}}=0$. Case (iv) is subdivided into node *i* in *A*_5_ and node *j* in *A*_1_, and node *i* in *A*_5_ and node *j* in (*A* − *A*_1_), and the improvement $\alpha_{A_{1}\to (A-A_{1})}=0$. Based on the above observations, the probability $P_{Link-Com}^{\left(A_{1}\to A\right)}$ that *k* captured nodes are in *A*_1_ is derived as follows.

(20) $$\left(\begin{array}{l} P_{Link-Com}^{\left(A_{1}\to A\right)}\approx \frac{1}{n^{2}}\cdot (\overset{\mathrm{case}\;(i)\text{:}\;\mathrm{node}\;i\;\mathrm{in}\;A_{1}\;\mathrm{node}\;j\;\mathrm{in}\;A_{5}}{\overset{︷}{\sum_{l=q}^{m}{\left(1-{\left(1-\alpha_{A_{1}\to A_{1}}\cdot \frac{m}{\left|S\right|}\right)}^{k}\right)}^{l}\cdot \frac{P_{Share}^{\left(1\right)}(l)}{\sum_{l=q}^{m}P_{Share}^{\left(1\right)}(l)}}}+\overset{\begin{array}{l} \mathrm{case}\;(\mathrm{ii})\text{:}\;\mathrm{node}\;i\;\mathrm{in}\;A_{2}\\ \mathrm{node}\;j\;\mathrm{in}\;A-(A_{1}\cup A_{2}) \end{array}}{\overset{︷}{\begin{array}{c} 0\\ \left(∵\alpha_{A_{1}\to A_{2}}=0\right) \end{array}}}+\overset{\begin{array}{l} \mathrm{case}\;(\mathrm{iii})\text{:}\;\mathrm{node}\;i\;\mathrm{in}\;A_{3}\\ \mathrm{node}\;j\;\mathrm{in}\;A-(A_{1}\cup A_{3}) \end{array}}{\overset{︷}{\begin{array}{c} 0\\ \left(∵\alpha_{A_{1}\to A_{3}}=0\right) \end{array}}}+\\ \overset{\mathrm{case}\;(\mathrm{iv})\text{:}\;\mathrm{node}\;i\;\mathrm{in}\;A_{5}\;\mathrm{node}\;j\;\mathrm{in}\;A}{\overset{︷}{\overset{i\;\mathrm{in}\;A_{5}\;j\;\mathrm{in}\;A_{1}}{\overset{︷}{\sum_{l=q}^{m}{\left(1-{\left(1-\alpha_{A_{1}\to A_{1}}\cdot \frac{m}{\left|S\right|}\right)}^{k}\right)}^{l}\cdot \frac{P_{Share}^{\left(1\right)}(l)}{\sum_{l=q}^{m}P_{Share}^{\left(1\right)}(l)}}}+\overset{i\;\mathrm{in}\;A_{5}\;j\;\mathrm{in}\;(A-A_{1})}{\overset{︷}{\begin{array}{c} 0\\ \left(∵\alpha_{A_{1}\to (A-A_{1})}=0\right) \end{array}}}}})=\frac{2n_{1}n_{5}}{n^{2}}\cdot \left(\sum_{l=q}^{m}{\left(1-{\left(1-\frac{\left(n_{1}+1\right)}{2n_{1}}\cdot \frac{m}{\left|S\right|}\right)}^{k}\right)}^{l}\cdot \frac{P_{Share}^{\left(1\right)}(l)}{\sum_{l=q}^{m}P_{Share}^{\left(1\right)}(l)}\right) \end{array}\right.$$

Same as deriving $P_{Link-Com}^{\left(A_{1}\to A\right)}$, we could derive $P_{Link-Com}^{\left(A_{1}\to B\right)}\approx \frac{2n_{1}n_{6}}{n^{2}}\cdot$ $\left(\sum_{l=q}^{m}{\left(1-{\left(1-\frac{\left(n_{1}+1\right)}{2n_{1}}\frac{m}{\left|S\right|}\right)}^{k}\right)}^{l}\cdot \frac{P_{Share}^{\left(1\right)}(l)}{\sum_{l=q}^{m}P_{Share}^{\left(1\right)}(l)}\right)$ and $P_{Link-Com}^{\left(A_{1}\to C\right)}\approx \frac{2n_{1}n_{7}}{n^{2}}\cdot \left(\sum_{l=q}^{m}{\left(1-{\left(1-\frac{\left(n_{1}+1\right)}{2n_{1}}\frac{m}{\left|S\right|}\right)}^{k}\right)}^{l}\right.$.$\left.\frac{P_{Share}^{\left(1\right)}(l)}{\sum_{l=q}^{m}P_{Share}^{\left(1\right)}(l)}\right)$. Then, the $P_{Link-Com}^{\left(A_{1}\to G\right)}$ is calculated as follows.

(21) $$\left\{\begin{array}{l} P_{Link-Com}^{\left(A_{1}\to G\right)}=P_{Link-Com}^{\left(A_{1}\to A\right)}+P_{Link-Com}^{\left(A_{1}\to B\right)}+P_{Link-Com}^{\left(A_{1}\to C\right)}\\ =\frac{2n_{1}(n_{5}+n_{6}+n_{7})}{n^{2}}\cdot \left(\sum_{l=q}^{m}{\left(1-{\left(1-\frac{\left(n_{1}+1\right)}{2n_{1}}\frac{m}{\left|S\right|}\right)}^{k}\right)}^{l}\cdot \frac{P_{Share}^{\left(1\right)}(l)}{\sum_{l=q}^{m}P_{Share}^{\left(1\right)}(l)}\right) \end{array}\right.$$

For calculating $P_{Link-Com}^{\left(A_{2}\to G\right)}$ and $P_{Link-Com}^{\left(A_{3}\to G\right)}$, we first derive $P_{Link-Com}^{\left(A_{2}\to A\right)}$ in Equation (22). By the same argument, we also have $P_{Link-Com}^{\left(A_{2}\to B\right)}$, $P_{Link-Com}^{\left(A_{3}\to A\right)}$, and $P_{Link-Com}^{\left(A_{3}\to C\right)}$. Then, $P_{Link-Com}^{\left(A_{2}\to G\right)}$ and $P_{Link-Com}^{\left(A_{3}\to G\right)}$ are derived in Equations (23) and (24).

(22) $$\left(\begin{array}{l} P_{Link-Com}^{\left(A_{2}\to A\right)}\approx \frac{1}{n^{2}}\cdot (\overset{\mathrm{case}\;(i)\text{:}\;\mathrm{node}\;i\;\mathrm{in}\;A_{1}\;\mathrm{node}\;j\;\mathrm{in}\;A_{5}}{\overset{︷}{\sum_{l=q}^{m}{\left(1-{\left(1-\alpha_{A_{2}\to A_{1}}\cdot \frac{m}{\left|S\right|}\right)}^{k}\right)}^{l}\cdot \frac{P_{Share}^{\left(1\right)}(l)}{\sum_{l=q}^{m}P_{Share}^{\left(1\right)}(l)}}}+\overset{\begin{array}{l} \mathrm{case}\;(\mathrm{ii})\text{:}\;\mathrm{node}\;i\;\mathrm{in}\;A_{2}\\ \mathrm{node}\;j\;\mathrm{in}\;A-(A_{1}\cup A_{2}) \end{array}}{\overset{︷}{\sum_{l=q}^{m}{\left(1-{\left(1-\alpha_{A_{2}\to A_{2}}\cdot \frac{m}{\left|S\right|}\right)}^{k}\right)}^{l}\cdot \frac{P_{Share}^{\left(1\right)}(l)}{\sum_{l=q}^{m}P_{Share}^{\left(1\right)}(l)}}}\\ +\overset{\begin{array}{l} \mathrm{case}\;(\mathrm{iii})\text{:}\;\mathrm{node}\;i\;\mathrm{in}\;A_{3}\\ \mathrm{node}\;j\;\mathrm{in}\;A-(A_{1}\cup A_{3}) \end{array}}{\overset{︷}{\overset{i\;\mathrm{in}\;A_{3}\;j\;\mathrm{in}\;A_{2}}{\overset{︷}{\sum_{l=q}^{m}{\left(1-{\left(1-\alpha_{A_{2}\to A_{2}}\cdot \frac{m}{\left|S\right|}\right)}^{k}\right)}^{l}\cdot \frac{P_{Share}^{\left(1\right)}(l)}{\sum_{l=q}^{m}P_{Share}^{\left(1\right)}(l)}}}+\overset{i\;\mathrm{in}\;A_{3}\;j\;\mathrm{in}\;A_{5}}{\overset{︷}{\begin{array}{c} 0\\ \left(∵\alpha_{A_{2}\to A_{3}}=0\right) \end{array}}}}}+\overset{\mathrm{case}\;(\mathrm{iv})\text{:}\;\mathrm{node}\;i\;\mathrm{in}\;A_{5}\;\mathrm{node}\;j\;\mathrm{in}\;A}{\overset{︷}{\overset{i\;\mathrm{in}\;A_{5}\;j\;\mathrm{in}\;A_{1}}{\overset{︷}{\sum_{l=q}^{m}{\left(1-{\left(1-\alpha_{A_{2}\to A_{1}}\cdot \frac{m}{\left|S\right|}\right)}^{k}\right)}^{l}\cdot \frac{P_{Share}^{\left(1\right)}(l)}{\sum_{l=q}^{m}P_{Share}^{\left(1\right)}(l)}}}}}\\ \overset{\mathrm{case}\;(\mathrm{iv})\text{:}\;\mathrm{node}\;i\;\mathrm{in}\;A_{5}\;\mathrm{node}\;j\;\mathrm{in}\;A}{\overset{︷}{+\overset{i\;\mathrm{in}\;A_{5}\;j\;\mathrm{in}\;A_{2}}{\overset{︷}{\sum_{l=q}^{m}{\left(1-{\left(1-\alpha_{A_{2}\to A_{2}}\cdot \frac{m}{\left|S\right|}\right)}^{k}\right)}^{l}\cdot \frac{P_{Share}^{\left(1\right)}(l)}{\sum_{l=q}^{m}P_{Share}^{\left(1\right)}(l)}}}+\overset{i\;\mathrm{in}\;A_{5}\;j\;\mathrm{in}\;(A_{3}\cup A_{5})}{\overset{︷}{\begin{array}{c} 0\\ \left(∵\alpha_{A_{2}\to (A_{3}\cup A_{5})}=0\right) \end{array}}}}})\\ =\frac{2n_{1}n_{5}}{n^{2}}\cdot \left(\sum_{l=q}^{m}{\left(1-{\left(1-\frac{m}{\left|S\right|}\right)}^{k}\right)}^{l}\cdot \frac{P_{Share}^{\left(1\right)}(l)}{\sum_{l=q}^{m}P_{Share}^{\left(1\right)}(l)}\right)+\frac{2n_{2}(n_{3}+n_{5})}{n^{2}}\cdot \left(\sum_{l=q}^{m}{\left(1-{\left(1-\frac{\left(n_{2}+1\right)}{2n_{2}}\frac{m}{\left|S\right|}\right)}^{k}\right)}^{l}\cdot \frac{P_{Share}^{\left(1\right)}(l)}{\sum_{l=q}^{m}P_{Share}^{\left(1\right)}(l)}\right) \end{array}\right.$$

(23) $$\left\{\begin{array}{l} P_{Link-Com}^{\left(A_{2}\to G\right)}=P_{Link-Com}^{\left(A_{2}\to A\right)}+P_{Link-Com}^{\left(A_{2}\to B\right)}\\ \approx \frac{2n_{1}n_{5}}{n^{2}}\cdot \left(\sum_{l=q}^{m}{\left(1-{\left(1-\frac{m}{\left|S\right|}\right)}^{k}\right)}^{l}\cdot \frac{P_{Share}^{\left(1\right)}(l)}{\sum_{l=q}^{m}P_{Share}^{\left(1\right)}(l)}\right)+\frac{2n_{2}(n_{3}+n_{5})}{n^{2}}\cdot \left(\sum_{l=q}^{m}{\left(1-{\left(1-\frac{\left(n_{2}+1\right)}{2n_{2}}\frac{m}{\left|S\right|}\right)}^{k}\right)}^{l}\cdot \frac{P_{Share}^{\left(1\right)}(l)}{\sum_{l=q}^{m}P_{Share}^{\left(1\right)}(l)}\right)\\ +\frac{2n_{1}n_{6}}{n^{2}}\cdot \left(\sum_{l=q}^{m}{\left(1-{\left(1-\frac{m}{\left|S\right|}\right)}^{k}\right)}^{l}\cdot \frac{P_{Share}^{\left(1\right)}(l)}{\sum_{l=q}^{m}P_{Share}^{\left(1\right)}(l)}\right)+\frac{2n_{2}(n_{4}+n_{6})}{n^{2}}\cdot \left(\sum_{l=q}^{m}{\left(1-{\left(1-\frac{\left(n_{2}+1\right)}{2n_{2}}\frac{m}{\left|S\right|}\right)}^{k}\right)}^{l}\cdot \frac{P_{Share}^{\left(1\right)}(l)}{\sum_{l=q}^{m}P_{Share}^{\left(1\right)}(l)}\right)\\ =\frac{2n_{1}(n_{5}+n_{6})}{n^{2}}\cdot \left(\sum_{l=q}^{m}{\left(1-{\left(1-\frac{m}{\left|S\right|}\right)}^{k}\right)}^{l}\cdot \frac{P_{Share}^{\left(1\right)}(l)}{\sum_{l=q}^{m}P_{Share}^{\left(1\right)}(l)}\right)+\frac{4n_{2}(n_{3}+n_{5})}{n^{2}}\cdot \left(\sum_{l=q}^{m}{\left(1-{\left(1-\frac{\left(n_{2}+1\right)}{2n_{2}}\frac{m}{\left|S\right|}\right)}^{k}\right)}^{l}\cdot \frac{P_{Share}^{\left(1\right)}(l)}{\sum_{l=q}^{m}P_{Share}^{\left(1\right)}(l)}\right) \end{array}\right.$$

(24) $$\left\{\begin{array}{l} P_{Link-Com}^{\left(A_{3}\to G\right)}=P_{Link-Com}^{\left(A_{3}\to A\right)}+P_{Link-Com}^{\left(A_{3}\to C\right)}\\ \approx \frac{2(n_{1}n_{5}+n_{2}n_{3}+n_{2}n_{5})}{n^{2}}\cdot \left(\sum_{l=q}^{m}{\left(1-{\left(1-\frac{m}{\left|S\right|}\right)}^{k}\right)}^{l}\cdot \frac{P_{Share}^{\left(1\right)}(l)}{\sum_{l=q}^{m}P_{Share}^{\left(1\right)}(l)}\right)+\frac{2n_{3}n_{5}}{n^{2}}\cdot \left(\sum_{l=q}^{m}{\left(1-{\left(1-\frac{\left(n_{3}+1\right)}{2n_{3}}\frac{m}{\left|S\right|}\right)}^{k}\right)}^{l}\cdot \frac{P_{Share}^{\left(1\right)}(l)}{\sum_{l=q}^{m}P_{Share}^{\left(1\right)}(l)}\right)\\ +\frac{2n_{1}n_{7}}{n^{2}}\cdot \left(\sum_{l=q}^{m}{\left(1-{\left(1-\frac{m}{\left|S\right|}\right)}^{k}\right)}^{l}\cdot \frac{P_{Share}^{\left(1\right)}(l)}{\sum_{l=q}^{m}P_{Share}^{\left(1\right)}(l)}\right)+\frac{2n_{3}(n_{4}+n_{7})}{n^{2}}\cdot \left(\sum_{l=q}^{m}{\left(1-{\left(1-\frac{\left(n_{3}+1\right)}{2n_{3}}\frac{m}{\left|S\right|}\right)}^{k}\right)}^{l}\cdot \frac{P_{Share}^{\left(1\right)}(l)}{\sum_{l=q}^{m}P_{Share}^{\left(1\right)}(l)}\right)\\ =\frac{2n_{1}(n_{5}+n_{7})+2n_{2}(n_{3}+n_{5})}{n^{2}}\cdot \left(\sum_{l=q}^{m}{\left(1-{\left(1-\frac{m}{\left|S\right|}\right)}^{k}\right)}^{l}\cdot \frac{P_{Share}^{\left(1\right)}(l)}{\sum_{l=q}^{m}P_{Share}^{\left(1\right)}(l)}\right)+\frac{2n_{3}(n_{4}+n_{5}+n_{7})}{n^{2}}\cdot \left(\sum_{l=q}^{m}{\left(1-{\left(1-\frac{\left(n_{3}+1\right)}{2n_{3}}\frac{m}{\left|S\right|}\right)}^{k}\right)}^{l}\cdot \frac{P_{Share}^{\left(1\right)}(l)}{\sum_{l=q}^{m}P_{Share}^{\left(1\right)}(l)}\right) \end{array}\right.$$

For the case using $P_{Share}^{\left(1\right)}(l)$ only (a large portion $57/64\cdot P_{Share}^{\left(1\right)}(l)$ in Equation (4)), $P_{Share}^{\left(2\right)}(l)$ and $P_{Share}^{\left(3\right)}(l)$ are very small (note: choosing the values $n_{1}=n_{2}=n_{3}=n_{4}=n/8$ is a reasonable choice). Equations (25)–(27) show that the probabilities $P_{Link-Com}^{\left(A_{1}\to G\right)}$ and $P_{Link-Com}^{\left(A_{2}\to G\right)}$ of I^2^KPHC are lesser than those in I^2^KPDP and I^2^KPSP, and $P_{Link-Com}^{\left(A_{3}\to G\right)}$ is almost the same as $P_{Link-Com}^{\left(A_{3}\to G\right)}$ in I^2^KPDP and I^2^KPSP. □

(25) $$\left\{\begin{array}{l} P_{Link-Com}^{\left(A_{1}\to G\right)}\approx \frac{2n_{1}(n_{5}+n_{6}+n_{7})}{n^{2}}\cdot \left(\sum_{l=q}^{m}{\left(1-{\left(1-\frac{\left(n_{1}+1\right)}{2n_{1}}\frac{m}{\left|S\right|}\right)}^{k}\right)}^{l}\cdot \frac{P_{Share}^{\left(1\right)}(l)}{\sum_{l=q}^{m}P_{Share}^{\left(1\right)}(l)}\right)\\ =\frac{30}{64}\cdot \left(\sum_{l=q}^{m}{\left(1-{\left(1-\frac{\left(n_{1}+1\right)}{2n_{1}}\frac{m}{\left|S\right|}\right)}^{k}\right)}^{l}\cdot \frac{P_{Share}^{\left(1\right)}(l)}{\sum_{l=q}^{m}P_{Share}^{\left(1\right)}(l)}\right)<\frac{30}{64}\cdot \left(\sum_{l=q}^{m}{\left(1-{\left(1-\frac{m}{\left|S\right|}\right)}^{k}\right)}^{l}\cdot \frac{P_{Share}^{\left(1\right)}(l)}{\sum_{l=q}^{m}P_{Share}^{\left(1\right)}(l)}\right)\\ <\left(\sum_{l=q}^{m}{\left(1-{\left(1-\frac{m}{\left|S\right|}\right)}^{k}\right)}^{l}\cdot \frac{P_{Share}^{\left(1\right)}(l)}{\sum_{l=q}^{m}P_{Share}^{\left(1\right)}(l)}\right) \end{array}\right.$$

(26) $$\left\{\begin{array}{l} P_{Link-Com}^{\left(A_{2}\to G\right)}\approx \frac{2n_{1}(n_{5}+n_{6})}{n^{2}}\cdot \left(\sum_{l=q}^{m}{\left(1-{\left(1-\frac{m}{\left|S\right|}\right)}^{k}\right)}^{l}\cdot \frac{P_{Share}^{\left(1\right)}(l)}{\sum_{l=q}^{m}P_{Share}^{\left(1\right)}(l)}\right)+\frac{4n_{2}(n_{3}+n_{5})}{n^{2}}\cdot \left(\sum_{l=q}^{m}{\left(1-{\left(1-\frac{\left(n_{2}+1\right)}{2n_{2}}\frac{m}{\left|S\right|}\right)}^{k}\right)}^{l}\cdot \frac{P_{Share}^{\left(1\right)}(l)}{\sum_{l=q}^{m}P_{Share}^{\left(1\right)}(l)}\right)\\ =\frac{20}{64}\cdot \left(\sum_{l=q}^{m}{\left(1-{\left(1-\frac{m}{\left|S\right|}\right)}^{k}\right)}^{l}\cdot \frac{P_{Share}^{\left(1\right)}(l)}{\sum_{l=q}^{m}P_{Share}^{\left(1\right)}(l)}\right)+\frac{24}{64}\cdot \left(\sum_{l=q}^{m}{\left(1-{\left(1-\frac{\left(n_{2}+1\right)}{2n_{2}}\frac{m}{\left|S\right|}\right)}^{k}\right)}^{l}\cdot \frac{P_{Share}^{\left(1\right)}(l)}{\sum_{l=q}^{m}P_{Share}^{\left(1\right)}(l)}\right)\\ \approx \frac{20}{64}\cdot \left(\sum_{l=q}^{m}{\left(1-{\left(1-\frac{m}{\left|S\right|}\right)}^{k}\right)}^{l}\cdot \frac{P_{Share}^{\left(1\right)}(l)}{\sum_{l=q}^{m}P_{Share}^{\left(1\right)}(l)}\right)+\frac{24}{64}\cdot \frac{\left(n_{2}+1\right)}{2n_{2}}\cdot \left(\sum_{l=q}^{m}{\left(1-{\left(1-\frac{m}{\left|S\right|}\right)}^{k}\right)}^{l}\cdot \frac{P_{Share}^{\left(1\right)}(l)}{\sum_{l=q}^{m}P_{Share}^{\left(1\right)}(l)}\right)\\ \left(∵1-{\left(1-\frac{\left(n_{2}+1\right)}{2n_{2}}\frac{m}{\left|S\right|}\right)}^{k}\approx \frac{\left(n_{2}+1\right)}{2n_{2}}\times \left(1-{\left(1-\frac{\left(n_{2}+1\right)}{2n_{2}}\frac{m}{\left|S\right|}\right)}^{k}\right)\;(\mathrm{while}\;k\;\mathrm{is}\;\mathrm{reasonably}\;\mathrm{low}\;\mathrm{and}\;m\ll |S|\right)\\ \approx \frac{20}{64}\cdot \left(\sum_{l=q}^{m}{\left(1-{\left(1-\frac{m}{\left|S\right|}\right)}^{k}\right)}^{l}\cdot \frac{P_{Share}^{\left(1\right)}(l)}{\sum_{l=q}^{m}P_{Share}^{\left(1\right)}(l)}\right)+\frac{12}{64}\cdot \left(\sum_{l=q}^{m}{\left(1-{\left(1-\frac{m}{\left|S\right|}\right)}^{k}\right)}^{l}\cdot \frac{P_{Share}^{\left(1\right)}(l)}{\sum_{l=q}^{m}P_{Share}^{\left(1\right)}(l)}\right)\\ =\frac{32}{64}\cdot \left(\sum_{l=q}^{m}{\left(1-{\left(1-\frac{m}{\left|S\right|}\right)}^{k}\right)}^{l}\cdot \frac{P_{Share}^{\left(1\right)}(l)}{\sum_{l=q}^{m}P_{Share}^{\left(1\right)}(l)}\right)<\frac{2}{3}\cdot \left(\sum_{l=q}^{m}{\left(1-{\left(1-\frac{m}{\left|S\right|}\right)}^{k}\right)}^{l}\cdot \frac{P_{Share}^{\left(1\right)}(l)}{\sum_{l=q}^{m}P_{Share}^{\left(1\right)}(l)}\right) \end{array}\right.$$

(27) $$\left\{\begin{array}{l} P_{Link-Com}^{\left(A_{3}\to G\right)}\approx \frac{2n_{1}(n_{5}+n_{7})+2n_{2}(n_{3}+n_{5})}{n^{2}}\cdot \left(\sum_{l=q}^{m}{\left(1-{\left(1-\frac{m}{\left|S\right|}\right)}^{k}\right)}^{l}\cdot \frac{P_{Share}^{\left(1\right)}(l)}{\sum_{l=q}^{m}P_{Share}^{\left(1\right)}(l)}\right)+\frac{2n_{3}(n_{4}+n_{5}+n_{7})}{n^{2}}\cdot \left(\sum_{l=q}^{m}{\left(1-{\left(1-\frac{\left(n_{3}+1\right)}{2n_{3}}\frac{m}{\left|S\right|}\right)}^{k}\right)}^{l}\right.\\ \left.\cdot \frac{P_{Share}^{\left(1\right)}(l)}{\sum_{l=q}^{m}P_{Share}^{\left(1\right)}(l)}\right)=\frac{32}{64}\cdot \left(\sum_{l=q}^{m}{\left(1-{\left(1-\frac{m}{\left|S\right|}\right)}^{k}\right)}^{l}\cdot \frac{P_{Share}^{\left(1\right)}(l)}{\sum_{l=q}^{m}P_{Share}^{\left(1\right)}(l)}\right)+\frac{22}{64}\cdot \left(\sum_{l=q}^{m}{\left(1-{\left(1-\frac{\left(n_{3}+1\right)}{2n_{3}}\frac{m}{\left|S\right|}\right)}^{k}\right)}^{l}\cdot \frac{P_{Share}^{\left(1\right)}(l)}{\sum_{l=q}^{m}P_{Share}^{\left(1\right)}(l)}\right)\\ \approx (\frac{32}{64}+\frac{11}{64})\cdot \left(\sum_{l=q}^{m}{\left(1-{\left(1-\frac{m}{\left|S\right|}\right)}^{k}\right)}^{l}\cdot \frac{P_{Share}^{\left(1\right)}(l)}{\sum_{l=q}^{m}P_{Share}^{\left(1\right)}(l)}\right)\;(\mathrm{note}\text{:}\;\mathrm{use}\;\mathrm{the}\;\mathrm{approximation}\;\mathrm{in}\;\mathrm{derivng}\;\mathrm{Equation}\;(26))\\ =\frac{33}{64}\cdot \left(\sum_{l=q}^{m}{\left(1-{\left(1-\frac{m}{\left|S\right|}\right)}^{k}\right)}^{l}\cdot \frac{P_{Share}^{\left(1\right)}(l)}{\sum_{l=q}^{m}P_{Share}^{\left(1\right)}(l)}\right)\approx \frac{2}{3}\cdot \left(\sum_{l=q}^{m}{\left(1-{\left(1-\frac{m}{\left|S\right|}\right)}^{k}\right)}^{l}\cdot \frac{P_{Share}^{\left(1\right)}(l)}{\sum_{l=q}^{m}P_{Share}^{\left(1\right)}(l)}\right) \end{array}\right.$$

In the proposed I^2^KPHC, extra hash operations are required to share the common key between two nodes. The following evaluates the computation overhead in our I^2^KPHC. Similar to deriving the number of hash operations for I^2^KPSP, we use the approximation $P_{Share}^{\left(x\right)}(l)\approx P_{Share}^{\left(1\right)}(l)$ and ignore $P_{Share}^{\left(2\right)}(l)$ and $P_{Share}^{\left(3\right)}(l)$ (it is reasonable for $n_{1}=n_{2}=n_{3}=n_{4}=$n/8). We still consider four cases using $P_{Share}^{\left(1\right)}(l)$ in group *A* as deriving $P_{Share}^{\left(A\right)}(l)$: (i) node *i* in *A*_1_ and node *j* in *A*_5_, (ii) node *i* in *A*_2_ and node *j* in $A-(A_{1}\cup A_{2})$, (iii) node *i* in *A*_3_ and node *j* in $A-(A_{1}\cup A_{3})$, and (iv) node *i* in *A*_5_ and node *j* in *A*. Thus, the average number ${N^{\prime }}_{avg}^{\left(A\right)}$ of hash operations to share a common key is determined in Equation (28).


(28) $$\left\{\begin{array}{l} {N^{\prime }}_{avg}^{\left(A\right)}=\frac{1}{n^{2}}\cdot (\overset{\begin{array}{c} \mathrm{case}\;(i)\text{:}\\ \mathrm{node}\;i\;\mathrm{in}\;A_{1}\;\mathrm{node}\;j\;\mathrm{in}\;A_{5} \end{array}}{\overset{︷}{\sum_{j=1}^{n_{5}}\sum_{i=(n_{2}+n_{3}+n_{5}+1)}^{n}\left(i-j\right)}}+\overset{\begin{array}{c} \mathrm{case}\;(\mathrm{ii})\text{:}\\ \mathrm{node}\;i\;\mathrm{in}\;A_{2}\;\mathrm{node}\;j\;\mathrm{in}\;A-(A_{1}\cup A_{2}) \end{array}}{\overset{︷}{\sum_{j=1}^{n_{3}+n_{5}}\sum_{i=(n_{3}+n_{5}+1)}^{n_{2}+n_{3}+n_{5}}\left(i-j\right)}}+\overset{\mathrm{case}\;(\mathrm{iii})\text{:}\;\mathrm{node}\;i\;\mathrm{in}\;A_{3}\;\mathrm{node}\;j\;\mathrm{in}\;A-(A_{1}\cup A_{3})}{\overset{︷}{\overset{i\;\mathrm{in}\;A_{3}\;j\;\mathrm{in}\;A_{5}}{\overset{︷}{\sum_{j=1}^{n_{5}}\sum_{i=(n_{5}+1)}^{n_{3}+n_{5}}\left(i-j\right)}}+\overset{i\;\mathrm{in}\;A_{3}\;j\;\mathrm{in}\;A_{2}}{\overset{︷}{\sum_{i=(n_{5}+1)}^{n_{3}+n_{5}}\sum_{j=(n_{3}+n_{5}+1)}^{n_{2}+n_{3}+n_{5}}\left(j-i\right)}}}}\\ +\overset{\mathrm{case}\;(\mathrm{iv})\text{:}\;\mathrm{node}\;i\;\mathrm{in}\;A_{5}\;\mathrm{node}\;j\;\mathrm{in}\;A}{\overset{︷}{\overset{i\;\mathrm{in}\;A_{5}\;j\;\mathrm{in}\;A_{5}}{\overset{︷}{\sum_{i=1}^{n_{5}}\sum_{j=1}^{n_{5}}\left|i-j\right|}}+\overset{i\;\mathrm{in}\;A_{5}\;j\;\mathrm{in}\;(A-A_{5})}{\overset{︷}{\sum_{i=1}^{n_{5}}\sum_{j=(n_{5}+1)}^{n}\left(j-i\right)}}}})=\overset{\mathrm{case}\;(i)}{\overset{︷}{\frac{n_{1}(n+n_{2}+n_{3})}{2n^{2}}}}+\overset{\mathrm{case}\;(\mathrm{ii})}{\overset{︷}{\frac{n_{2}(n_{2}+n_{3}+n_{5})}{2n^{2}}}}+\overset{\mathrm{case}\;(\mathrm{iii})}{\overset{︷}{\frac{\left(n_{2}^{2}+n_{2}n_{3}+n_{3}^{2}+n_{3}n_{5}\right)}{2n^{2}}}}+\\ \overset{\mathrm{case}\;(\mathrm{iv})}{\overset{︷}{\frac{{n_{5}}^{3}-n_{5}}{3n^{2}}+\frac{(n_{1}+n_{2}+n_{3})n}{2n^{2}}}} \end{array}\right.$$

Similar to deriving ${N^{\prime }}_{avg}^{\left(A\right)}$, we can figure out ${N^{\prime }}_{avg}^{\left(B\right)}$ and ${N^{\prime }}_{avg}^{\left(C\right)}$. Finally, in *q*-composite I^2^KPHC, both nodes should apply the average hash operations $N_{I^{2}\mathrm{KPHC}}^{\left(x\right)}={N^{\prime }}_{avg}^{\left(x\right)}\times$ $\sum_{l=q}^{m}\left(l\times \frac{P_{Share}^{\left(1\right)}(l)}{\sum_{l=q}^{m}P_{Share}^{\left(1\right)}(l)}\right)$ in group *x* when establishing a secure link.

## 5. Comparison and Numerical Simulations

### 5.1. Comparison

A comparison of various types of pairwise key predistribution schemes including KP, HKP, and our three types of I^2^KP—I^2^KPDP, I^2^KPSP, and I^2^KPHC—is shown in Table 1. We compare these schemes by the following items: (i) the key ring size, (ii) intragroup and intergroup conditions, (iii) the resiliency against LNA, (iv) the improvement in resiliency using a hash chain, (v) the probability of compromising a link, and (vi) the number of hash operations for establishing a link. After that, we will give some numerical simulations for resiliency and computation overhead.

As illustrated in Table 1, there are two schemes using hash chains: HKP and I^2^KPHC. Although I^2^KPSP uses one hash function *f*(·), it is not based on a hash chain. The hash function in I^2^KPSP is used to separate the raw key and hashed key, such that the key ring sizes in intersected groups can be reduced. About the form of the key, the keys in sensors are the raw keys in KP, I^2^KPDP, and the intersected groups of I^2^KPSP. Other schemes store the hashed keys in their key rings. Our three I^2^KP schemes have large key ring sizes, but they satisfy I^2^-conditions (intragroup and intergroup conditions). I^2^KPDP and I^2^KPSP suffer with LNAs. I^2^KPHC uses the large ID for sensor nodes in intersected areas to tackle LNAs.

### 5.2. Numerical Simulation

The resiliency (i.e., 1–the probability of a compromised link) and the network connectivity are two major terms we use to evaluate the performance key predistribution for WSNs. As defined in the intragroup condition, the nodes in one group with a common sink node have a pairwise key predistribution ability like KP. On the other hand, the intergroup condition shows that the nodes in different groups are pairwise-disjointed for the key ring, and thus they do not have the same keys to establish secure links. This is why we do not evaluate the connectivity, since the intergroup nodes cannot connect with each other, and it is the same as KP in the intragroup. In simulations, we only show the resiliency performance. We compare I^2^KPDP, I^2^KPSP, and I^2^KPHC when the compromised nodes are in different locations to check whether they meet intragroup and intergroup conditions, and we also evaluate their resiliencies against NAs and LNAs.

#### 5.2.1. Resiliency

**Figure 8:**
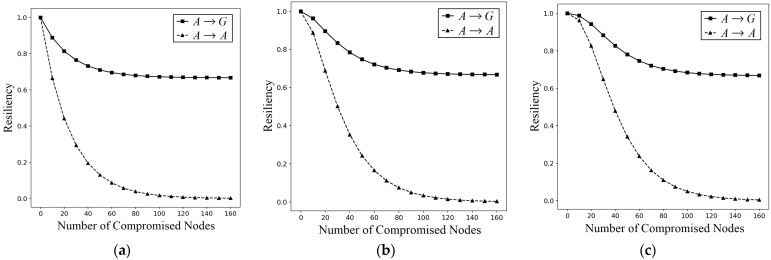
NA resiliency on the number of captured nodes for *q*-composite I^2^KPDP (I^2^KPSP) with (**a**) *q* = 1, (**b**) *q* = 2, and (**c**) *q* = 3.

Resiliency against NAs is defined as the fraction of uncompromised links when *k* nodes are compromised, i.e., the resiliency is “1–(the probability of compromised link when *k* nodes are captured)”. The resiliencies against NAs and LNAs of I^2^KPDP, I^2^KPSP, and I^2^KPHC are tested. In the numerical simulation, we use |S| = 1000, *m* = 40, *n* = 160, and $n_{1}=n_{2}=n_{3}=20$. I^2^KPSP has the same performance as I^2^KPDP, and thus we use I^2^KPDP for comparison only. The probability $P_{I^{2}\mathrm{KPDP}}^{\left(NA:\;A\to A\right)}=$ $\sum_{l=q}^{m}{\left(1-{\left(1-\frac{m}{\left|S\right|}\right)}^{k}\right)}^{l}\cdot \frac{P_{Share}^{\left(1\right)}(l)}{\sum_{l=q}^{m}P_{Share}^{\left(1\right)}(l)}$ is the same as $P_{\mathrm{KP}}^{\left(NA\right)}=P_{Link-Com}^{\left(A\right)}=\sum_{l=q}^{m}{\left(1-{\left(1-\frac{m}{\left|S\right|}\right)}^{k}\right)}^{l}\cdot \frac{P_{Share}^{\left(1\right)}(l)}{\sum_{l=q}^{m}P_{Share}^{\left(1\right)}(l)}$. Considering the effect on *G*, the probability is $P_{I^{2}\mathrm{KPDP}}^{\left(NA:\;A\to G\right)}\approx \frac{C_{k}^{n}+C_{k}^{n_{1}+n_{2}}+C_{k}^{n_{1}+n_{3}}}{3C_{k}^{n}}\cdot \left(\sum_{l=q}^{m}{\left(1-{\left(1-\frac{m}{\left|S\right|}\right)}^{k}\right)}^{l}\cdot \frac{P_{Share}^{\left(1\right)}(l)}{\sum_{l=q}^{m}P_{Share}^{\left(1\right)}(l)}\right)$ (see Equation (9)). We plot the resiliencies depending on the number of captured nodes in group *A* with the compromised links in *A* and *G*, respectively, for *q*-composite I^2^KPDP and I^2^KPSP. As illustrated in Figure 8a–c, the values of *q* are 1, 2, and 3, respectively. This figure shows clearly that the resiliency against NA is better when using our approach. This is because the *k* captured nodes in group *A* may be ineffective to compromise the links in other groups of *G*. The notation ( ■  A→G) in Figure 8 implies that the captured nodes in set *A* and the compromised links are located in the whole group G=(A∪B∪C). The notation ( ▲  A→A) implies that the captured nodes in set *A* and the compromised links are also located in the set *A*. The case ( ▲  A→A) is the same as applying KP in a single set, i.e., I^2^KPDP for the case ( ▲  A→A) is reduced as in conventional KP. For NAs, it is observed that I^2^KPDP (I^2^KPSP) with higher resiliency is really better than conventional KP. The above results also demonstrate that the intragroup condition is satisfied. Our I^2^KPDP (I^2^KPSP) is just KP for the intragroup case. On the other hand, the intergroup condition is also satisfied, since the nodes in $A-(A_{1}\cup A_{2}\cup A_{3})$ cannot be used for compromising the link in sets *B* and *C*. Thus, the resiliency of ( ■  A→G) is better than that of ( ▲  A→A).

**Figure 9:**
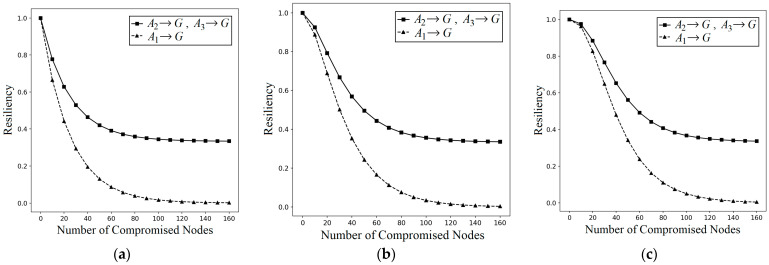
LNA resiliency on the number of captured nodes for *q*-composite I^2^KPDP (I^2^KPSP) with (**a**) *q* = 1, (**b**) *q* = 2, and (**c**) *q* = 3.

When applying LNAs on I^2^KPDP (I^2^KPDP), we have $P_{I^{2}\mathrm{KPDP}}^{\left(LNA:\;A_{1}\to G\right)}>P_{I^{2}\mathrm{KPDP}}^{\left(LNA:\;A_{2}\to G\right)}$ $(=P_{I^{2}\mathrm{KPDP}}^{\left(LNA:\;A_{3}\to G\right)})>P_{I^{2}\mathrm{KPDP}}^{\left(NA:\;A\to G\right)}$, implying that I^2^KPDP and I^2^KPSP cannot resist LNAs. Namely, an LNA is more severe than an NA. The $P_{I^{2}\mathrm{KPDP}}^{\left(LNA:\;A_{1}\to G\right)}$ is $\sum_{l=q}^{m}{\left(1-{\left(1-\frac{m}{\left|S\right|}\right)}^{k}\right)}^{l}\cdot \frac{P_{Share}^{\left(1\right)}(l)}{\sum_{l=q}^{m}P_{Share}^{\left(1\right)}(l)}$ and the $P_{I^{2}\mathrm{KPDP}}^{\left(LNA:\;A_{2}\to G\right)}$$\left(=P_{I^{2}\mathrm{KPDP}}^{\left(LNA:\;A_{3}\to G\right)}\right)$ is $\frac{2}{3}\left(\sum_{l=q}^{m}{\left(1-{\left(1-\frac{m}{\left|S\right|}\right)}^{k}\right)}^{l}\cdot \frac{P_{Share}^{\left(1\right)}(l)}{\sum_{l=q}^{m}P_{Share}^{\left(1\right)}(l)}\right)$ (see Equation (17)). Figure 9 shows the resiliencies of I^2^KPDP (I^2^KPSP) against LNAs when the captured nodes are in sets $A_{1}$, $A_{2}$, and $A_{3}$, respectively, as shown Figure 9a–c. Sets $A_{2}$ and $A_{3}$ have two intersected groups. If attackers intentionally compromise the nodes in $A_{1}$ with three intersected groups to aggravate the NA (actually, this is an LNA), I^2^KPDP (I^2^KPSP) does not have resiliency. Thus, it is observed that ( ▲  A1→G) is worse than ( ■  A2→G, A3→G). The above shows that I^2^KPDP (I^2^KPSP) cannot resist LNAs.

**Figure 10:**
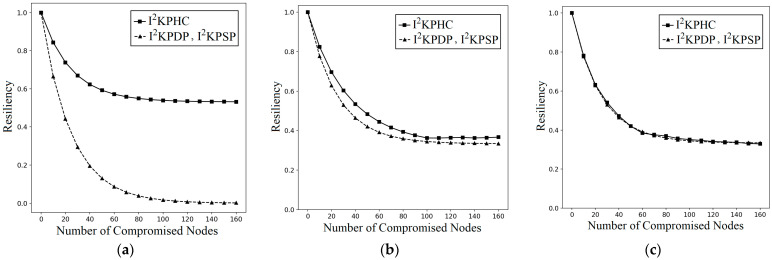
LNA resiliency on the number of captured nodes for the 1-composite I^2^KPDP (I^2^KPSP) and I^2^KPHC when the *k* captured nodes are in the intersected group: (**a**) *A*_1_; (**b**) *A*_2_; (**c**) *A*_3_.

On the other hand, for I^2^KPHC, we have $P_{I^{2}\mathrm{KPHC}}^{\left(LNA:\;A_{1}\to G\right)}<P_{I^{2}\mathrm{KPDP}}^{\left(LNA:\;A_{1}\to G\right)}$, $P_{I^{2}\mathrm{KPHC}}^{\left(LNA:\;A_{1}\to G\right)}<P_{I^{2}\mathrm{KPDP}}^{\left(LNA:\;A_{1}\to G\right)}$, and $P_{I^{2}\mathrm{KPHC}}^{\left(LNA:\;A_{3}\to G\right)}\approx P_{I^{2}\mathrm{KPDP}}^{\left(LNA:\;A_{3}\to G\right)}$ (see Theorem 4), and this implies that I^2^KPHC enhances the resilience against LNAs. Figure 10a–c demonstrates the probabilities $P_{I^{2}\mathrm{KPHC}}^{\left(LNA:\;A_{i}\to G\right)}\;\mathrm{and}\;P_{I^{2}\mathrm{KPDP}}^{\left(LNA:\;A_{i}\to G\right)}$ of single-composite schemes for *I* = 1, 2, and 3, respectively. Figure 10 compares the LNA resiliency between I^2^KPDP (I^2^KPSP) and I^2^KPHC. The key selection of I^2^KPHC (Figure 7a) is consistent with the LNA resiliency of I^2^KPHC when the compromised nodes are in $A_{1}$, $A_{2}$, and $A_{3}$. As shown in Figure 10a,b, the ( ■  I2KPHC) has higher LNA resiliency than ( ▲  I2KPDP, I2KPSP) when compromised nodes are located in $A_{1}$ and $A_{2}$. Furthermore, I^2^KPHC has more LNA resiliency when compromised nodes are in area $A_{1}$ since the hashed keys in $A_{1}$ are $h_{A}^{j}(Key),$ $\;j\in [n_{2}+n_{3}+n_{5}+1,\;n].$ When captured nodes are in $A_{3}$, the hashed keys $h_{A}^{j}(Key),$ $j\in [n_{5}+1,\;n_{3}+n_{5}]$ have small *j* values. Thus, I^2^KPHC has almost the same LNA resiliency compared with I^2^KPDP (I^2^KPSP) (see Figure 10c).

#### 5.2.2. Computation Overhead

For the three I^2^KP models, there is no need to use hash operations for I^2^KPDP. Other schemes need hash operations when sharing a common key between nodes. Although I^2^KPSP uses a hash function, this function *f*(·) is just used to reduce the key ring size. I^2^KPHC is based on the hash chain, and thus this scheme has more hash operations than I^2^KPSP. As described in the above, we use |S| = 1000, *m* = 40, *n* = 160, and $n_{1}=n_{2}=n_{3}=20$ (i.e., =*n*/8) in this numerical simulation. From Equation (16), we have $N_{avg}^{\left(x\right)}=17/32$, *x*∈{*A*, *B*, *C*}, for I^2^KPSP. Thus, in *q*-composite I^2^KPSP, the average number of hash operation is 1.048, 1.399, and 1.829 for *q* = 1, 2, and 3, respectively.

Next, we adopting the key-chain length *L* to save energy consumption overhead and test I^2^KPHC. We use *L* = 1, *L* = 8, *L* = 16, *L* = 24, and *L* = 32, and the values *L*_1_ = *L*_2_ = *L*_3_ = *L*_4_ = *L*/8. Because *L* = *L*_1_ + *L*_2_ + *L*_3_ + *L*_5_, *L* = *L*_1_ + *L*_2_ + *L*_4_ + *L*_6_ and *L* = *L*_1_ + *L*_2_ + *L*_4_ + *L*_7_, we have *L*_5_ = *L*_6_ = *L*_7_ = (5 *L*)/8. Then, for *L* = 8, 16, 24, and 32, we have *L*_1_ = *L*_2_ = *L*_3_ = *L*_4_ = 1, 2, 3, and 4, and *L*_5_ = *L*_6_ = *L*_7_ = 5, 10, 15, and 20, respectively. Based on these values, we summarize the average number of hash computations of *q*-composite I^2^KPHC (*q* = 1, 2, and 3), ${N^{\prime }}_{avg}^{\left(x\right)}$ (from Equation (28)) in Table 2. As described in Section 4.3, when calculating ${N^{\prime }}_{avg}^{\left(x\right)}$, the values of *n* and $n_{i},\;1\leq i\leq 7,$ should be replaced by *L* and $L_{i}$. This table shows that the average number of hash computations remains reasonable when *L* ≤ 32. Note that with *L* = 1, we exactly have conventional KP, where sensor nodes store raw keys but not hashed keys. Thus, the value ${N^{\prime }}_{avg}^{\left(x\right)}$ is zero.

## 6. Conclusions

We can use an MWSN to implement a scalable WSN and gradually deploy a large-scale WSN as the number of sensor nodes increases. Therefore, it is indeed necessary to study pairwise key predistribution in MWSNs. In this paper, we propose three types of I^2^KP: I^2^KPDP, I^2^KPSP, and I^2^KPHC. These three schemes all meet the intragroup and intergroup conditions. Each has its own advantages and disadvantages, and they are all suitable for the MWSN environment. We discuss two attacks, NAs and LNAs, based on the amount of information known to the attacker. In actual use, we can choose one of these three types of I^2^KP for the MWSN according to the resiliency against variants of node capture attacks, the link connectivity, the required hash operations, and the key ring size of the sensor node. Finally, the three types of I^2^KP not only provide security but also retain the advantages of using an MWSN.

## Figures and Tables

**Table 1:** Comparison of various KP schemes.

Scheme	Key Ring Size	I^2^-Cond.	LNA Resiliency	Improvement Against LNA	Prob. of Compromised Link	Num. of Hash Operations
KP	*m*	NO	NO	*–*	${P_{\left(\mathrm{KP}\right)}}^{\#2}$	*–*
HKP	*m*	NO	NO	*–*	${P_{\left(\mathrm{HKP}\right)}}^{\#3}$	${N_{\mathrm{HKP}}^{\left(x\right)}}^{\#6}$
I^2^KPDP	*m*/2*m*/3*m*	YES	NO	*–*	${P_{{(I}^{2}\mathrm{KPDP})}}^{\#4}$	*–*
I^2^KPSP	*m*/(*m* + 2)/(*m* + 3)	YES	NO	*–*	${P_{{(I}^{2}\mathrm{KPSP})}}^{\#4}$	${N_{I^{2}\mathrm{KPSP}}^{\left(x\right)}}^{\#7}$
I^2^KPHC	*m*/2*m*/3*m*	YES	YES	${\alpha_{A_{i}\to A_{j}}}^{\#1}$	${P_{{(I}^{2}\mathrm{KPHC})}}^{\#5}$	${N_{I^{2}\mathrm{KPHC}}^{\left(x\right)}}^{\#8}$

$\#1:\mathrm{improvent}\;\mathrm{for}\;\mathrm{LNA}\;\alpha_{A_{i}\to A_{j}}=\left(n_{i}+1\right)/\left(2n_{i}\right),\;0,\;\mathrm{or}\;1\;(\mathrm{Lemma}\;2)\;\#2:\;P_{\left(\mathrm{KP}\right)}=P_{Link-Com}^{\left(x\right)}=\sum_{l=q}^{m_{x}}{\left(1-{\left(1-\frac{m_{x}}{\left|S_{x}\right|}\right)}^{k}\right)}^{l}\cdot \frac{P_{Share}^{\left(x\right)}(l)}{P_{Link-Est}^{\left(x\right)}}$; $\#3:\;P_{\left(\mathrm{HKP}\right)}=P_{Link-Com}^{\left(x\right)}=\sum_{l=q}^{m_{x}}{\left(1-{\left(1-\frac{n+1}{2n}\frac{m_{x}}{\left|S_{x}\right|}\right)}^{k}\right)}^{l}\cdot \frac{P_{Share}^{\left(x\right)}(l)}{P_{Link-Est}^{\left(x\right)}}\;\text{\#}4\text{:}\;P_{{(I}^{2}\mathrm{KPDP})}=P_{{(I}^{2}\mathrm{KPSP})}=P_{Link-Com}^{\left(A_{i}\to G\right)}\;(\mathrm{Theorem}\;3)$; $\#5:\;P_{{(I}^{2}\mathrm{KPHC})}=P_{Link-Com}^{\left(A_{i}\to G\right)}\;(\mathrm{Theorem}\;4)\;\text{\#}6:N_{\mathrm{HKP}}^{\left(x\right)}=\frac{\left(n^{2}-1\right)}{6n}\times \sum_{l=q}^{m}\left(l\times P_{Share}^{\left(1\right)}(l)/\sum_{l=q}^{m}P_{Share}^{\left(1\right)}(l)\right)$; $\text{\#}7:N_{I^{2}\mathrm{KPSP}}^{\left(x\right)}=N_{avg}^{\left(x\right)}\times \sum_{l=q}^{m}\left(l\times P_{Share}^{\left(1\right)}(l)/\sum_{l=q}^{m}P_{Share}^{\left(1\right)}(l)\right)\;\text{\#}8:N_{I^{2}\mathrm{KPHC}}^{\left(x\right)}={N^{\prime }}_{avg}^{\left(x\right)}\times \sum_{l=q}^{m}\left(l\times P_{Share}^{\left(1\right)}(l)/\sum_{l=q}^{m}P_{Share}^{\left(1\right)}(l)\right)$.

**Table 2:** Average number of hash operations for I^2^KPHC.

L	${N^{\prime }}_{avg}^{\left(x\right)}$	$N_{I^{2}KPHC}^{\left(x\right)}$		
		*q* = 1	*q* = 2	*q* = 3
1	0	0	0	0
8	0.487	0.961	1.283	1.677
16	0.500	0.986	1.317	1.722
24	0.504	0.995	1.328	1.737
32	0.507	0.999	1.334	1.744

## Data Availability

All the data are contained within the article.
